# Systems analysis of the CO_2_ concentrating mechanism in cyanobacteria

**DOI:** 10.7554/eLife.02043

**Published:** 2014-04-29

**Authors:** Niall M Mangan, Michael P Brenner

**Affiliations:** 1 School of Engineering and Applied Sciences and Kavli Institute for Bionano Science and Technology, Harvard University, Cambridge, United States; Weizmann Institute for Science, Israel

**Keywords:** cyanobacteria, carboxysomes, carbon fixation, systems modeling, other

## Abstract

Cyanobacteria are photosynthetic bacteria with a unique CO_2_ concentrating mechanism (CCM), enhancing carbon fixation. Understanding the CCM requires a systems level perspective of how molecular components work together to enhance CO_2_ fixation. We present a mathematical model of the cyanobacterial CCM, giving the parameter regime (expression levels, catalytic rates, permeability of carboxysome shell) for efficient carbon fixation. Efficiency requires saturating the RuBisCO reaction, staying below saturation for carbonic anhydrase, and avoiding wasteful oxygenation reactions. We find selectivity at the carboxysome shell is not necessary; there is an optimal non-specific carboxysome shell permeability. We compare the efficacy of facilitated CO_2_ uptake, CO_2_ scavenging, and ${\text{HCO}}_{3}^{-}$ transport with varying external pH. At the optimal carboxysome permeability, contributions from CO_2_ scavenging at the cell membrane are small. We examine the cumulative benefits of CCM spatial organization strategies: enzyme co-localization and compartmentalization.

**DOI:** http://dx.doi.org/10.7554/eLife.02043.001

## eLife digest

Cyanobacteria are microorganisms that live in water and, like plants, they capture energy from the sun to convert carbon dioxide into sugars and other useful compounds. This process—called photosynthesis—releases oxygen as a by-product. Cyanobacteria were crucial in making the atmosphere of the early Earth habitable for other organisms, and they created the vast carbon-rich deposits that now supply us with fossil fuels. Modern cyanobacteria continue to sustain life on Earth by providing oxygen and food for other organisms, and researchers are trying to bioengineer cyanobacteria to produce alternative, cleaner, fuels.

Understanding how cyanobacteria can be as efficient as possible at harnessing sunlight to ‘fix’ carbon dioxide into carbon-rich molecules is an important step in this endeavor. Carbon dioxide can readily pass through cell membranes, so instead cyanobacteria accumulate molecules of bicarbonate inside their cells. This molecule is then converted back into carbon dioxide by an enzyme found in specials compartments within cells called carboxysomes. The enzyme that fixes the carbon is also found in the carboxysomes. However, several important details in this process are not fully understood.

Here, Mangan and Brenner further extend a mathematical model of the mechanism that cyanobacteria use to concentrate carbon dioxide in order to explore the factors that optimize carbon fixation by these microorganisms. Carbon fixation is deemed efficient when there is more carbon dioxide in the carboxysome than the carbon-fixing enzyme can immediately use (which also avoids wasteful side-reactions that use oxygen instead of carbon dioxide). However, there should not be too much bicarbonate, otherwise the enzyme that converts it to carbon dioxide is overwhelmed and cannot take advantage of the extra bicarbonate.

Mangan and Brenner's model based the rates that carbon dioxide and bicarbonate could move in and out of the cell, and the rates that the two enzymes work, on previously published experiments. The model varied the location of the enzymes (either free in the cell or inside a carboxysome), and the rate at which carbon dioxide and bicarbonate could diffuse in and out of the carboxysome (the carboxysome's permeability). Mangan and Brenner found that containing the enzymes within a carboxysome increased the concentration of carbon dioxide inside the cell by an order of magnitude. The model also revealed the optimal permeability for the carboxysome outer-shell that would maximize carbon fixation.

In addition to being of interest to researchers working on biofuels, if the model can be adapted to work for plant photosynthesis, it may help efforts to boost crop production to feed the world’s growing population.

**DOI:** http://dx.doi.org/10.7554/eLife.02043.002

## Introduction

Intracellular compartments are critical for directing and protecting biochemical reactions. One of the simplest and most striking known examples of compartmentalization are the carboxysomes (Cannon et al., 2001; Yeates et al., 2008) of cyanobacteria and other autotrophic proteobacteria (Savage et al., 2010; Rosgaard et al., 2012). These small, 100–200 nm compartments separate the principal reaction of the Calvin cycle, the RuBisCO catalyzed fixation of carbon dioxide (CO_2_) into 3-phosphoglycerate, from the rest of the cell (Cannon et al., 1991). CO_2_ and oxygen (O_2_) competitively bind as substrates of RuBisCO, and the reaction with O_2_ produces phosphoglycolate, a waste product which must be recycled by the cell (Jordan & Ogren, 1981; Tcherkez et al., 2006; Savir et al., 2010). To maximize carboxylation and minimize oxygenation, the carboxysome is believed to act as a diffusion barrier to CO_2_ (Reinhold et al., 1989; Dou et al., 2008). There is much interest in the design and function of such compartments and whether they can be used to enhance carbon fixation in other organisms such as plants or to improve reaction rates in other metabolic systems (Ducat & Silver, 2012; Agapakis et al., 2012; Papapostolou & Howorka, 2009; Frank et al., 2013). Increased efficiency of biochemical reactions will lead to better yield in bioengineered bacterial systems, creating new possibilities for production of high-value products such as biofuels. Enhancing carbon fixation in plants or other organisms could lead to increased carbon sequestration, or crop yield.

**Figure 1.**
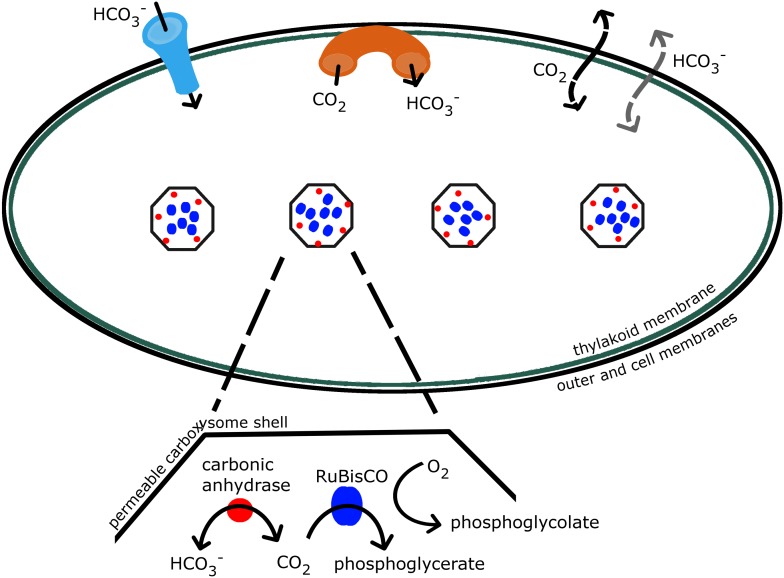
Schematic of the CCM in cyanobacteria. Outer and cell membranes (in black), as well as, thylakoid membranes where the light reactions take place (in green) are treated together. Carboxysomes are shown as four hexagons evenly spaced along the centerline of the cell. The model treats a spherically symmetric cell, with one carboxysome at the center. Active ${\text{HCO}}_{3}^{-}$ transport into the cell is indicated (in light blue), as well as active conversion from CO_2_ to ${\text{HCO}}_{3}^{-}$, sometimes called ‘facilitated uptake’ or ‘scavenging’, at membranes (in orange). Both CO_2_ and ${\text{HCO}}_{3}^{-}$ can leak in and out of the cell, with CO_2_ leaking out much more readily. Both species passively diffuse across the carboxysome shell. Carbonic anhydrase (red) and RuBisCO (blue) are contained in the carboxysomes and facilitate reactions as shown. **DOI:** http://dx.doi.org/10.7554/eLife.02043.003

The concentrating mechanism in cyanobacteria relies on the interaction of a number of well characterized components, as shown in Figure 1, transferring inorganic carbon from outside the cell into cytosol and carboxysomes (Allen, 1984; Badger & Price, 2003; Kaplan & Reinhold, 1999; Price et al., 2007). Due to this mechanism, inorganic carbon concentration is elevated well above 200–300 *μ*M, the CO_2_ concentration required for saturating the RuBisCO. Additionally a high CO_2_ concentration increases the ratio of CO_2_ to O_2_ so that carboxylation dominates over oxygenation. Concentrations of 20–40 mM inorganic carbon, up to 4000-fold higher than external levels, have been observed (Kaplan & Reinhold, 1999; Woodger et al., 2005; Sultemeyer et al., 1995; Price et al., 1998). The bilipid outer and cell membranes are highly permeable to small uncharged molecules such as CO_2_ (Missner et al., 2008; Gutknecht et al., 1977), so instead the cell primarily accumulates the charged and less membrane soluble bicarbonate (${\text{HCO}}_{3}^{-}$) (Volokita et al., 1984; Price & Badger, 1989). Active transporters, both constitutive and inducible, bring ${\text{HCO}}_{3}^{-}$ into the cell (Price et al., 2008, 2004; Omata et al., 1999), and mechanisms exist to actively convert CO_2_ to ${\text{HCO}}_{3}^{-}$ at the thylakoid and cell membrane (Price et al., 2008; Maeda et al., 2002; Shibata et al., 2001). Once it passively diffuses into the carboxysome, ${\text{HCO}}_{3}^{-}$ is rapidly brought into equilibrium with CO_2_ by the enzyme carbonic anhydrase, resulting in the production of CO_2_ near RuBisCO. Carbonic anhydrase is known to be localized on the interior side of the carboxysome shell (Yeates et al., 2008; Cannon et al., 1991; Cot et al., 2008; Long et al., 2008). The carboxysome shell must be permeable enough to allow ${\text{HCO}}_{3}^{-}$ and 3-phosphoglycerate to readily diffuse in and out. The function of this system and its ability to concentrate inorganic carbon depends on the interplay between these various molecular components. Without a model, flux measurements cannot determine the components relative roles in enhancing the CO_2_ concentration in the carboxysome. To date, it has not been possible to directly measure the partitioning of the internal carbon concentration in the cytosol and carboxysomes. We wish to characterize the distribution of internal carbon. Visualizations of the location of the carboxysomes with fluorescent microscopy in *Synechococcus elongatus* PCC7942 demonstrated that the carboxysomes are evenly spaced along the centerline of the cell, (Savage et al., 2010), raising the question of how spatial organization, beyond simple partitioning, changes the efficacy of the system.

The goal of this study is to further develop a mathematical model of the CCM (Reinhold et al., 1989; Fridlyand et al., 1996; Reinhold et al., 1991) that uses recent experimental progress on the CCM to untangle the relative roles of the different molecular components, and predict the region of parameter space where efficient carbon fixation occurs. We are considering conditions where CO_2_ is limiting (15*μM* external inorganic carbon) and, for the moment, ignore other biological pressures. In this context, efficient carbon fixation requires two conditions: First, the CO_2_ concentration must be high enough that RuBisCO is saturated, and the competitive reaction with O_2_ is negligible. We emphasize that for the oxygenation reaction to be negligible the CO_2_ concentration should be higher than needed to merely saturate RuBisCO. Secondly, the carbonic anhydrase within the carboxysome must be unsaturated, so that extra energy isn't wasted transporting unused ${\text{HCO}}_{3}^{-}$ into the cell.

Examination of the system performance with varying expression levels of ${\text{HCO}}_{3}^{-}$ transporters, carboxysome permeability, and conversion from CO_2_ to ${\text{HCO}}_{3}^{-}$, reveals a parameter window where these conditions are simultaneously satisfied. We comment on the relation of this window to measured carbon pools, carbon fixation rates, and ${\text{HCO}}_{3}^{-}$ transporters. We find that the ${\text{HCO}}_{3}^{-}$ concentration in the cytosol is constant across the cell, set by the ${\text{HCO}}_{3}^{-}$ transport and leakage rates, and depends very little on the carboxysome permeability. The carboxysome permeability does, however, set how the CO_2_ is partitioned between the carboxysome and cytosol. At optimal carboxysome permeability, ${\text{HCO}}_{3}^{-}$ diffusion into the carboxysome is fast enough to supply inorganic carbon for fixation, but the rate of CO_2_ leakage out of the carboxysome is low. We explore the fluxes from CO_2_ facilitated uptake and scavenging with varying ratios of external CO_2_ and ${\text{HCO}}_{3}^{-}$. Finally we discuss the proportion the carbon concentration comes from different methods of spatial organization such as co-localization, encapsulation, and spatial location of carboxysomes. Concentration of carbonic anhydrase increases the maximum rate of reaction for carbonic anhydrase per volume, causing carbonic anhydrase to saturate at a higher level of ${\text{HCO}}_{3}^{-}$, and achieve an order of magnitude higher local CO_2_ concentrations. Encapsulation of the reactions in an optimally permeable carboxysome shell achieves another order of magnitude of CO_2_ concentration.

### Reaction diffusion model

We present our mathematical model, which captures all aspects of the CCM as described above. This model is an expansion of previously developed models (Reinhold et al., 1989; Fridlyand et al., 1996; Reinhold et al., 1991). Our three dimensional model of the CCM solves for both the ${\text{HCO}}_{3}^{-}$ and CO_2_ concentration throughout a spherical cell. We solve this model numerically and analytically at steady state for three different spatial organizations of carbonic anhydrase and RuBisCO in the cell (See Figure 6): enzymes distributed evenly throughout the cell, enzymes localized to the center of the cell but not encapsulated (as they would be on a scaffold), enzymes encapsulated in a carboxysome. We compare the effects of these scenarios in the discussion section, and for now consider a spherical cell of radius R_b_ = 0.5 *μm* with a single spherical carboxysome of radius R_c_ = 50 *nm* containing RuBisCO and carbonic anhydrase. Numerical computations are carried out with finite difference methods in MATLAB. The details of analytic solutions are given in the Supplementary file 1.

We include the effects of diffusion, active transport and leakage through the cell membrane, and reactions with carbonic anhydrase and RuBisCO. In the carboxysome (r < *R*_*c*_), the equations governing the ${\text{HCO}}_{3}^{-}$ and CO_2_, here H and C respectively, are

(1) $$\partial_{t}C=D\nabla^{2}C+R_{CA}-R_{Rub}$$

(2) $$\partial_{t}H=D\nabla^{2}H-R_{CA^{,}}$$

where here *D* is the diffusion constant, and R_CA_ is the carbonic anhydrase reaction, and R_Rub_ is the RuBisCO reaction. The carbonic anhydrase reaction follows reversible Michaelis–Menten kinetics (Kaplan & Reinhold, 1999; Price et al., 2007),

(3) $$R_{CA}\left(H,C\right)=\frac{V_{ba}K_{ca}H-V_{ca}K_{ba}C}{K_{ba}K_{ca}+K_{ca}H+KL_{ba}C},$$

where *V*_*ca*_ and *V*_*ba*_ are hydration and dehydration rates, proportional to the local carbonic anhydrase concentration. *K*_*ca*_ and *K*_*ba*_ are the concentration at which hydration and dehydration are half maximum. The RuBisCO reaction follows Michaelis–Menten kinetics with competitive binding with O_2_, $R_{Rub}=V_{\text{max}}C/\left(C+K_{m}\right)$, where $k_{m}=k_{m}^{\prime }(1+O/k_{i})$. Here *V*_*max*_ is the maximum rate of carbon fixation and K_m_ is the apparent half maximum concentration value, which has been modified to include competitive binding with O_2_, *O*. K_i_ is the dissociation constant of *O*_2_ with the RuBisCO and $K_{m}^{\prime }$ is the half maximum concentration with no *O*_2_ present. RuBisCO also requires ribulose-1,5-bisphosphate, the substrate which *CO*_2_ reacts with to produce 3-phosphoglycolate. Under CO_2_ limiting conditions it has been shown that there is sufficient ribulose-1,5-bisphosphate to saturate all RuBisCO active sites, and the reaction rates are independent of ribulose-1,5-bisphosphate concentrations (Mayo et al., 1989; Whitehead et al., 2014).

In the cytosol there is no carbonic anhydrase or RuBisCO activity, so R_CA_ = 0 and $R_{Rub}=0$, and there is only diffusion of CO_2_ and ${\text{HCO}}_{3}^{-}$. We do not include the natural, but slow, interconversion of CO_2_ and ${\text{HCO}}_{3}^{-}$ in the cytosol. This assumption is a good one given that the ${\text{HCO}}_{3}^{-}$ concentration is known to be held out of equilibrium in the cell (Volokita et al., 1984; Price & Badger, 1989). In agreement with this experimental observation, we find that all the other processes effecting the concentration of ${\text{HCO}}_{3}^{-}$ in the cytosol happen much faster than the natural interconversion.

Boundary conditions prescribe the inorganic carbon fluxes into the cell and the diffusion across the carboxysome boundary. We treat the inorganic carbon fluxes at cell and thylakoid membranes together. At this cell boundary, there is passive leakage of both CO_2_ and ${\text{HCO}}_{3}^{-}$: the velocity of CO_2_ across the cell membrane, $k_{m}^{c}$ is about 1000-fold higher than that of ${\text{HCO}}_{3}^{-}$, $k_{m}^{H}$, due to the lower permeability of the membrane to charged molecules. To account for active import of ${\text{HCO}}_{3}^{-}$, we combine the total ${\text{HCO}}_{3}^{-}$ flux, *j*_*c*_, from all ${\text{HCO}}_{3}^{-}$ transporters. These transporters include BCT1 (encoded by cpm), which is thought to be powered by ATP; and BicA and SbtA which are thought to be symporters between ${\text{HCO}}_{3}^{-}$ and Na^+^, driven by the highly controlled electrochemical gradient for Na^+^ (Price et al., 2008, 2004; Omata et al., 1999). Additionally, there are two complexes NDH-1_3_ and NDH-1_4_ responsible for converting CO_2_ to ${\text{HCO}}_{3}^{-}$. This conversion is thought to either decrease CO_2_, creating a gradient across the membranes and ‘facilitating uptake’ of CO_2_, or ‘scavenge’ CO_2_ which has escaped from the carboxysome. These are localized to the thylakoid and possibly the plasma membrane. They have been linked to the photosynthetic linear and cyclic electron transport chain (Price et al., 2008; Maeda et al., 2002; Shibata et al., 2001). It has been proposed that electron transport drives the formation of local alkaline pockets where CO_2_ more rapidly converts to ${\text{HCO}}_{3}^{-}$. We more simply describe the conversion with a maximal reaction rate *α*, and concentration of half maximal activity of *K*_*α*_. Combining these effects, the boundary condition setting diffusive flux of ${\text{HCO}}_{3}^{-}$ and CO_2_ at the cell membrane is

(4) $$D\partial_{r}C=-\frac{\alpha C_{cytosol}}{K_{\alpha }+C_{cytosol}}+k_{m}^{C}\left(C_{out}-C_{cytosol}\right)$$

(5) $$D\partial_{r}H=j_{c}H_{out}+\frac{\alpha C_{cytosol}}{K_{\alpha }+C_{cytosol}}+k_{m}^{H}\left(H_{out}-H_{cytosol}\right)$$

where the subscript *cytosol* and *out* indicate we are taking the concentration immediately inside and outside the cell boundary respectively. The diffusion constant times partial derivatives with respect to the radial coordinate, *r*, define the diffusive flux at the membrane.

The carboxysome shell is composed of proteins with a radius R_c_ ≈ 50 nm. As of yet, there have been no direct measurements of the carboxysome permeability to small molecules. Using the carboxysome geometry, we can calculate an upper bound for the diffusive velocity across the carboxysome shell, which is directly related to the carboxysome permeability. Crystal structures (Yeates et al., 2008; Cheng et al., 2008; yeates et al., 2007) show the surface has approximately N_pores_ = 4800 small pores with radius $r_{pore}\approx 0.35$ nm, and thickness *l* = 1.8 nm. If *k*_*c*_ is the characteristic velocity that small molecules pass through the shell, these numbers imply the upper bound for diffusive transport $k_{c}<\frac{\pi r_{pore}^{2}D}{4\pi R_{c}^{2}l}\left(N_{pores}\right)\approx 0.02\frac{cm}{s}$. This calculation is done by taking the probability that a molecule will encounter a pore on the carboxysome shell $\left(\frac{N_{pores}\times \text{pore surface area}}{\text{carboxysome surface area}}\right)$ and multipling it by the speed a small molecule will diffuse through the length of the pore (*D/l*). Since it does not take into account any charge effects, which would add an additional energy barrier, it is an upper bound. Although there has been much speculation that the positively charged pores might enhance diffusion of negatively charged ${\text{HCO}}_{3}^{-}$ (Yeates et al., 2008; Dou et al., 2008; Cheng et al., 2008), here we explore the simplest assumption, that both ${\text{HCO}}_{3}^{-}$ and CO_2_ have the same permeability. Namely, the boundary conditions at the carboxysome shell are

(6) $$D\partial_{r}C=k_{c}\left(C_{cytosol}-C_{carboxysome}\right)$$

(7) $$D\partial_{r}H=k_{c}(H_{cytosol}-H_{carboxysome}).$$

We will vary *k*_*c*_ (henceforth called carboxysome permeability, although it is a velocity) within our model and see that there is a range of *k*_*c*_ where the CCM is effective even with *k*_*c*_ identical for CO_2_ and ${\text{HCO}}_{3}^{-}$.

## Results

### Analysis of model: Finding functional parameter space

Now that we have defined our model, we wish to find the range of parameters where efficient carbon fixation occurs. In what follows, we fix the enzymatic rates, cell membrane permeability, and diffusion constant as reported in the literature (Jordan & Ogren, 1981; Missner et al., 2008; Gutknecht et al., 1977; Heinhorst et al., 2006) (see Table 1 and Table 2). Note that full analytic solutions are available in Supplementary file 1 sections S3 and S4, so the effect of varying other parameters can be analyzed. We consider the efficacy of the CCM as a function of *j*_*c*_, the flux of ${\text{HCO}}_{3}^{-}$ into the cell, *k*_*c*_, the carboxysome permeability, and the parameters (α, *K*_α_) governing the *CO*_*2*_ facilitated uptake mechanism. Both *α* and *j*_*c*_ can be regulated by the organism and vary depending on environmental conditions, whereas the carboxysome permeability, *k*_*c*_, is the parameter with the largest uncertainty and debate (Cannon et al., 2001; Yeates et al., 2008; Cheng et al., 2008).

**Table 1.** Parameter values chosen for main set of simulations, unless otherwise indicated **DOI:** http://dx.doi.org/10.7554/eLife.02043.005

Parameter	Definition	Value	Reference
*H*_*out*_	concentration of bicarbonate outside the cell	14 μM*	(Price et al., 2008)
*C*_*out*_	concentration of carbon dioxide outside of cell	0.14 μM*	(Price et al., 2008)
D	diffusion constant of small molecules, CO_2_ and ${\text{HCO}}_{3}^{-}$	10^−5^ $\frac{cm^{2}}{s}$	(Fridlyand et al., 1996)
$k_{m}^{c}$	permeability of cell membrane to CO_2_	0.3 $\frac{cm}{s}$	(Missner et al., 2008; Gutknecht et al., 1977)
$k_{m}^{H}$	permeability of cell membrane to ${\text{HCO}}_{3}^{-}$	$3\times 10^{-4}\frac{cm}{s}$	(Missner et al., 2008; Gutknecht et al., 1977)
*R*_*c*_	radius of carboxysome	5×10^−6^ cm	(Cheng et al., 2008; Schmid et al., 2006)
*R*_*b*_	radius of bacteria	5 × 10^−5^ cm	(Savage et al., 2010)
*j*_*c*_	${\text{HCO}}_{3}^{-}$ transport rate resulting in 30mM cytosolic ${\text{HCO}}_{3}^{-}$ pool	0.6 $\frac{cm}{s}$*	calculated here
$k_{c}$	optimal carboxysome permeability	10^−3^ $\frac{cm}{s}$*	calculated here
$V_{cell}$	cell volume	$5.2\times 10^{-10}\mu L$	calculated
$SA_{cell}$	cell surface area	$3\times 10^{-8}cm^{2}$	calculated

* these parameters are varied in the text, but these values are use unless noted otherwise.

**Table 2.** Table comparing enzymatic rates (Woodger et al., 2005; Sultemeyer et al., 1995; Heinhorst et al., 2006) **DOI:** http://dx.doi.org/10.7554/eLife.02043.006

Enzyme reaction	active sites	$k_{cat}$ $\left[\frac{1}{s}\right]$	$V_{\max}$ in ‘cell’ $\left[\frac{\mu M}{s}\right]$	$V_{\max}$ in carboxysome $\left[\frac{\mu M}{s}\right]$	$K_{1/2}$ [μM]
carbonic anhydrase hydration	80	8 × 10^4^	8.8 × 10^3^	1.5 × 10^7^	3.2 × 10^3^
carbonic anhydrase dehydration	80	4.6 × 10^4^	1.5 × 10^4^	8.8 × 10^6^	9.3 × 10^3^
RuBisCO carboxylation	2160	26	178	1.8 × 10^5^	270

$V_{\max}$ in cell and carboxysome refer to the volumetric reaction rate assuming the enzymes are distributed throughout the entire cell or only carboxysome. *V*_*ba*_ (*V*_*max*_ for carbonic anhydrase dehydration) is estimated by assuming *K*_*eq*_ = 5 and using parameters found in (Heinhorst et al., 2006). *V*_*ca*_ is *V*_*max*_ for carbonic anhydrase hydration. Similarly, *K*_*ba*_, and *K*_*ca*_ are $K_{1/2}$ for dehydration and hydration respectively.

**Figure 2.**
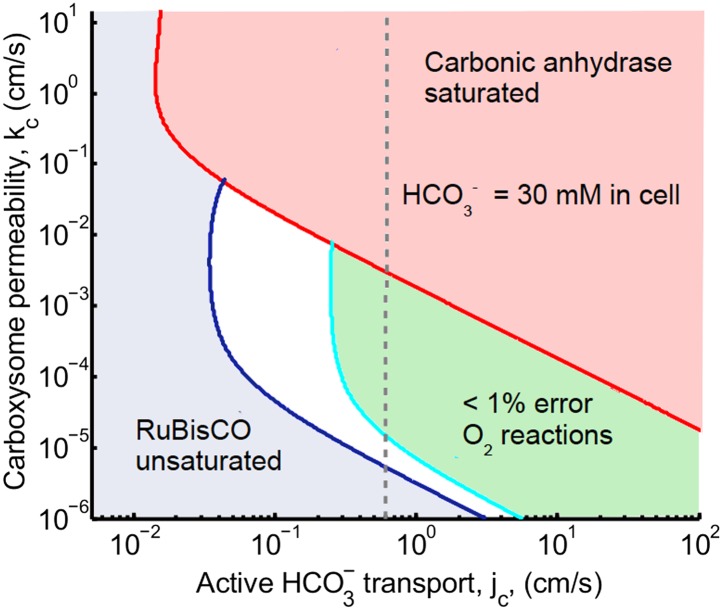
Phase space for ${\text{HCO}}_{3}^{-}$ transport, *j*_*c*_, and carboxysome permeability *k*_c_. Plotted are the parameter values at which the CO_2_ concentration reaches some critical value. The left most line (dark blue) indicates for what values of *j*_*c*_ and *k*_*c*_ the CO_2_ concentration in the carboxysome would half-saturate RuBisCO (*K*_*m*_). The middle line (light blue) indicates the parameter values which would result in a CO_2_ concentration where 99% of all RuBisCO reactions are carboxylation reactions and only 1% are oxygenation reactions when O_2_ concentration is 260 *μM*. The right most (red) line indicates the parameter values which result in carbonic anyhdrase saturating. Here α = 0, so there is no CO_2_ scavenging or facilitated uptake. The dotted line (grey) shows the *k*_*c*_ and *j*_*c*_ values, where the ${\text{HCO}}_{3}^{-}$ concentration in the cytosol is 30 *mM*. The ${\text{HCO}}_{3}^{-}$ concentration in the cytosol does not vary appreciably with *k*_*c*_ in this parameter regime, and reaches 30 *mM* at $j_{c}\approx 0.6\frac{cm}{s}$. All other parameters, such as reaction rates are held fixed and the value can be found in the Table 1 and Table 2. **DOI:** http://dx.doi.org/10.7554/eLife.02043.007

For any given pair of *k*_*c*_ and *j*_*c*_, we ask whether the CO_2_ concentrating mechanism is effective, using the criteria of saturating RuBisCO, reducing oxidation reactions, and not increasing the ${\text{HCO}}_{3}^{-}$ concentration beyond carbonic anhydrase saturation. Our central result is presented in Figure 2, which shows the range of *k*_*c*_ and *j*_*c*_ where these conditions are met, assuming that there is no facilitated uptake, *α* = 0. The blue shaded region shows where RuBisCO is unsaturated, and the red shaded region shows where carbonic anhydrase is saturated. There is a crescent shaped region between these regions, where the CCM is effective according to our criteria. In the white region oxygenation reactions happen at a rate of greater than 1%. In the green shaded region oxygenation reactions occur at a rate of less than 1%. Within the white and green regions the CO_2_ concentration in the carboxysome varies greatly.

**Figure 2—figure supplement 1.**
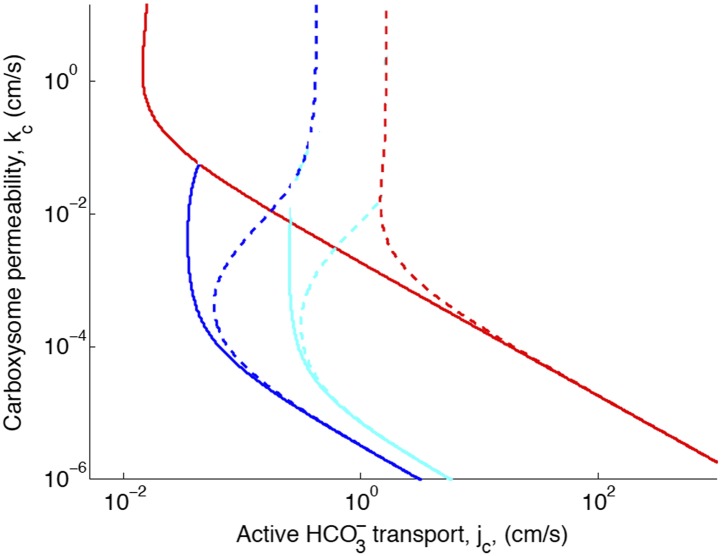
Phase space for ${\text{HCO}}_{3}^{-}$ transport and carboxysome permeability. Solid lines show lines of constant CO_2_ concentration in the carboxysome for $D_{c}=1e-5\frac{cm^{2}}{s}$, or the diffusion constant of small molecule in water. Dashed lines show the same lines of constant CO_2_ concentration, bur for $D_{c}=1e-7\frac{cm^{2}}{s}$, or the diffusion constant of a small molecule in a 60% sucrose solution. **DOI:** http://dx.doi.org/10.7554/eLife.02043.008

**Figure 2—figure supplement 2.**
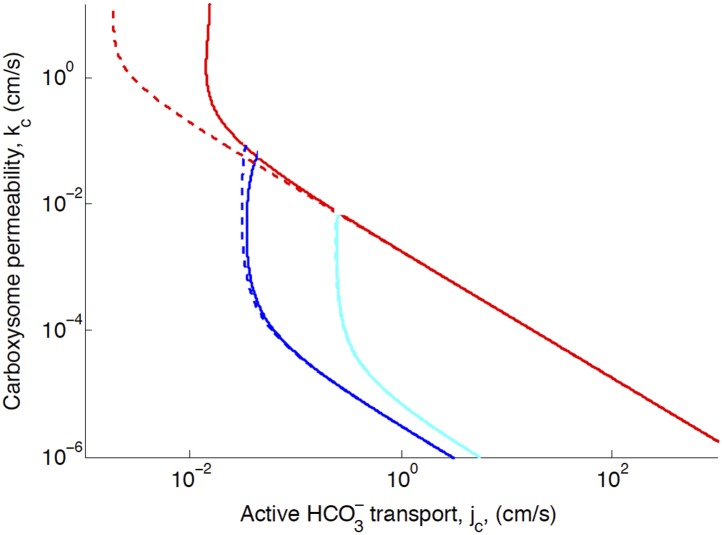
Phase space for ${\text{HCO}}_{3}^{-}$ transport, *j*_*c*_, and carboxysome permeability, *k*_*c*_. Plotted are the parameter values at which CO_2_ concentration reaches some critical value. The left most line (dark blue) indicates for what values of *j*_*c*_ and *k*_c_ the CO_2_ concentration in the carboxysome would saturate RuBisCO. The middle line (light blue) indicates the parameter values which would result in a CO_2_ concentration where 99% of all RuBisCO reactions are carboxylation reactions and only 1% are oxygenation reactions when O_2_ concentration is 260 *µM*. The right most (red) line indicates the parameter values which result in carbonic anyhdrase saturating. Here $\alpha =0\frac{cm}{s}$ (solid lines) and $\alpha =0\frac{cm}{s}$ (dashed line), showing the effect of CO_2_ scavenging or facilitated uptake on the phase space. All other parameters, such as reaction rates are held fixed and the value can be found Table 1. **DOI:** http://dx.doi.org/10.7554/eLife.02043.009

The lines dividing the regions in Figure 2 are lines of constant carboxysomal CO_2_ concentration in *j*_*c*_ and *k*_*c*_ parameter space. The dark blue line is where CO_2_ = K_m_, the CO_2_ concentration for half-maximum RuBisCO reactions. The light blue line indicates parameter values resulting in the CO_2_ concentration (C_99%_) where rate of oxygenation reactions is 1% for O_2_ concentration of 260 *μ*M. Varying carboxysome permeability, *k*_*c*_ values, require more or less HCO_3_ transport, *j*_*c*_, to achieve the same carboxysomal CO_2_ concentration.

We can calculate an amplification factor for the C_99%_ carboxysomal CO_2_ concentration as $A_{c}=\frac{C_{carboxysome}}{C_{out}+H_{out}}=133$. Any combination of *j*_*c*_ and *k*_*c*_ which produce C = C_99%_, make 133 times more CO_2_ available in the carboxysome than there is total inorganic carbon outside the cell. Generally, increasing ${\text{HCO}}_{3}^{-}$ transport, below the carbonic anhydrase saturation point results in higher CO_2_ concentration in the carboxysome.

### Varying ${\text{HCO}}_{3}^{-}$ transport saturates enzymes

**Figure 3.**
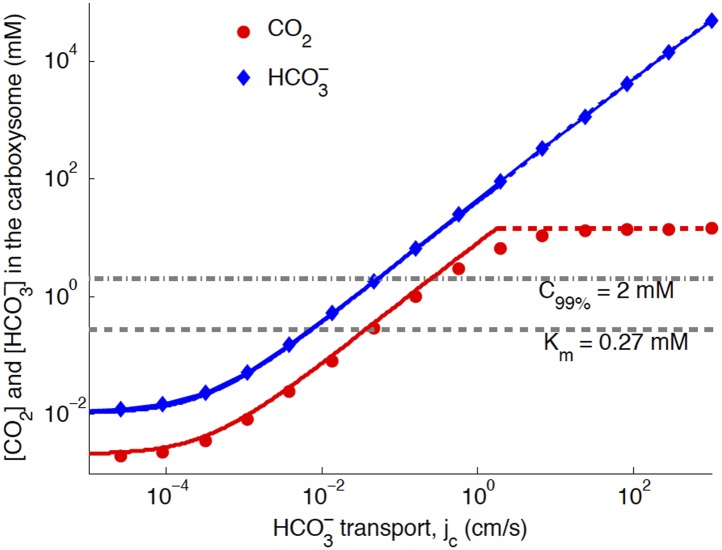
Numerical solution (diamonds and circles) and analytic solutions (carbonic anhydrase unsaturated, solid lines, and saturated, dashed lines) correspond well. ${\text{HCO}}_{3}^{-}$ transport is varied, and all other system parameters are held constant. The CO_2_ concentration above which RuBisCO is saturated is *K*_*m*_ (grey dashed line). The CO_2_ concentration where the oxygen reaction error rate will be 1% is C_99%_ (grey dash-dotted line). The transition between carbonic anyhdrase being unsatruated and saturated happens where the two analytic solutions meet (where the dashed and solid red lines meet). Below a critical value of transport, $j_{c}\approx 10^{-3}\frac{cm}{s}$ the level of transport is lower than the ${\text{HCO}}_{3}^{-}$ leaking through the cell membrane. A value of $k_{c}=10^{-3}\text{ cm}/\text{s}$ for the carboxysome permeability was used for these calculations. **DOI:** http://dx.doi.org/10.7554/eLife.02043.010

**Figure 4.**
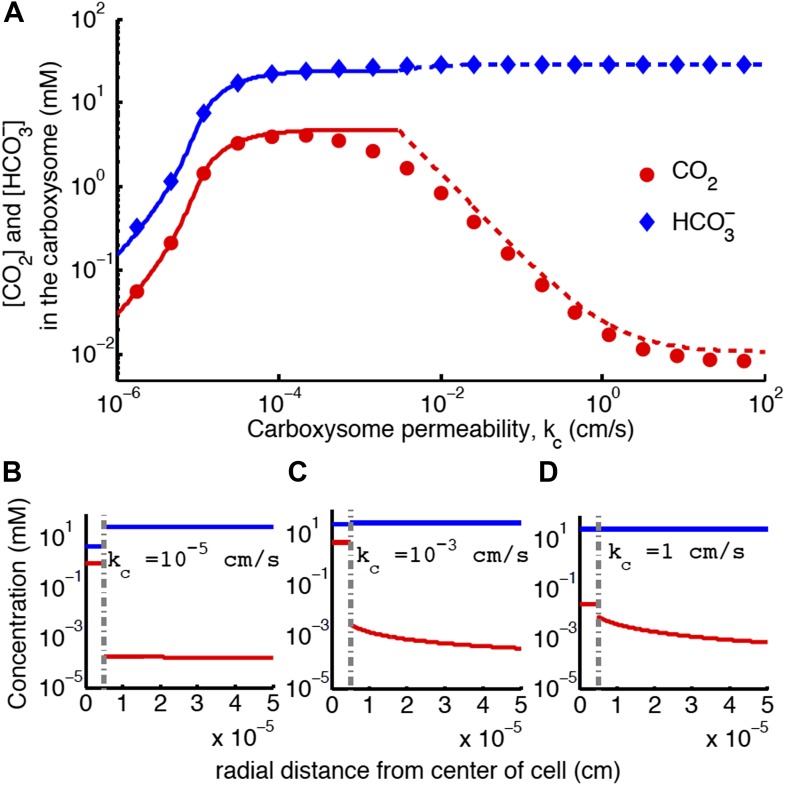
Concentration of CO_2_ in the carboxysome with varying carboxysome permeability (**A**). Numerical solution (diamonds and circles) and analytic solutions (carbonic anhydrase unsaturated, solid lines, and saturated, dashed lines) correspond well. On all plots CO_2_ (red circle) < ${\text{HCO}}_{3}^{-}$ (blue diamond). Concentration in the cell along the radius, *r*, with carboxysome permeability $k_{c}=10^{-5}\frac{cm}{s}$ (**B**), $k_{c}=10^{-3}\frac{cm}{s}$ (**C**), $k_{c}=1\frac{cm}{s}$ (**D**). Grey dotted lines in (**B**), (**C**), (**D**) indicate location of the carboxysome shell boundary. The transition from low CO_2_ at high permeability (**D**) to maximum CO_2_ concentration at optimal permeability (**C**) occurs at $k_{c}^{*}=\frac{D}{R_{c}}=2\frac{cm}{s}$. At low carboxysome permeability (**B**) ${\text{HCO}}_{3}^{-}$ diffusion into the carboxysome is slower than consumption. For all subplots $\alpha =0\frac{cm}{s}$ and $j_{c}=0.6\frac{cm}{s}$. Qualitative results remain the same with varying *j*_*c*_, increasing *α* will increase the gradient of CO_2_ across the cell as CO_2_ is converted to ${\text{HCO}}_{3}^{-}$ at the cell membrane. **DOI:** http://dx.doi.org/10.7554/eLife.02043.012

The basic physics of the phase diagram Figure 2 follows from examining how CO_2_ and ${\text{HCO}}_{3}^{-}$ in the carboxysome change as *j*_*c*_ is varied. Figure 3 shows the response to varying *j*_*c*_, with $k_{c}=10^{-3}\frac{cm}{s}$ (the optimal value in Figure 4).

**Figure 3—figure supplement 1.**
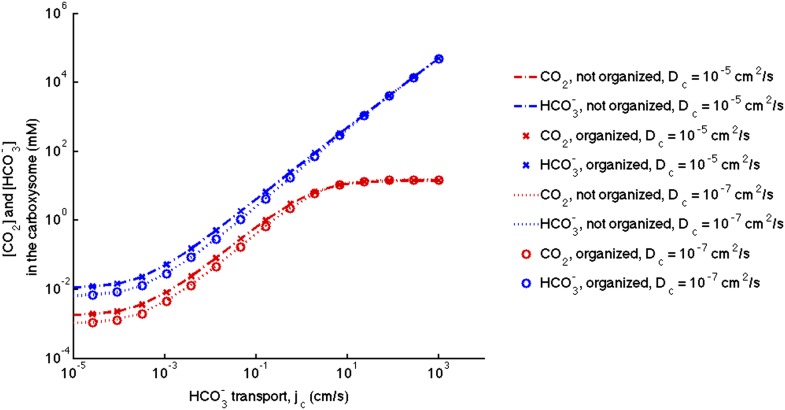
No effect of localizing carbonic anhydrase to the shell of the carboxysome. We assume the same amount of carbonic anhydrase and RuBisCO activity for each simulation and compare the case with the enzymes evenly distributed throughout the carboxysome to the case where the carbonic anhydrase is localized to the inner carboxysome shell. The (-.-) lines are for no organization and (x) for localization with $D_{c}=10^{-5}\frac{cm^{2}}{s}$. The (…) lines are for no organization and (o) are for localization with $D_{c}=10^{-7}\frac{cm^{2}}{s}$. **DOI:** http://dx.doi.org/10.7554/eLife.02043.011

When *j*_*c*_ is low, the ratio of CO_2_ and ${\text{HCO}}_{3}^{-}$ is constant, set by the chemical equilibrium at a given pH. In this case the rate of the carbonic anhydrase reaction is much faster than diffusion within the carboxysome, so that $V_{ba}K_{ca}H=V_{ca}K_{ba}C$. Unlike the uncatalyzed interconversion of CO_2_ and ${\text{HCO}}_{3}^{-}$ in the cytosol, carbonic anhydrase brings the concentrations in the carboxysome to equilibrium very quickly. The chemical equilibrium is $K_{eq}=H/C=(K_{ba}V_{ca})/\left(K_{ca}V_{ba}\right)\approx 5$, for pH around 7 [Heinhorst et al., 2006; DeVoe & Kistiakowsky, 1961)], so that ${\text{HCO}}_{3}^{-}$ > CO_2_ in the carboxysome. Increased pH would increase *K*_*eq*_ and the proportion of ${\text{HCO}}_{3}^{-}$, while decreased pH would decrease *K*_*eq*_ and the proportion of ${\text{HCO}}_{3}^{-}$. Such variations do not substantially effect the subsequent discussion and mechanisms, although they will change the absolute values of CO_2_ concentration in the carboxysome.

The *K*_*m*_ dashed line in Figure 3 shows the CO_2_ level above which RuBisCO reaction is saturated: this gives the RuBisCO saturated (blue) boundary in Figure 2. We have similarly marked the concentration C_99%_ where there is a 1% oxygen reaction error rate with a dash-doted line.

At higher levels, the CO_2_ concentration no longer increases with increasing *j*_*c*_, because the carbonic anhydrase is saturated. The saturated regime occurs in Figure 3 when $H_{carboxysome}>K_{ba}$, so that increasing $H_{carboxysome}$ (controlled directly by *j*_*c*_) no longer increases the rate of production of $C_{carboxysome}$. This transition from unsaturated to saturated carbonic anhydrase defines the line for the carbonic anhydrase saturated region in Figure 2.

### Carboxysome permeability has optimal value

For each line of constant concentration in Figure 2 there is an optimal permeability value, where the least ${\text{HCO}}_{3}^{-}$ transport is required to achieve the same CO_2_ concentration. The optimal permeability value shifts downward with increasing CO_2_ concentration (compare light and dark blue curves). For *C*_*99%*_ the optimal permeability is $k_{c}=10^{-3}\frac{cm}{s}$, below the calculated upper bound: $k_{c}<0.02\frac{cm}{s}$ obtained above from the carboxysome structure. To further understand the effect of permeability, we examine the CO_2_ concentration in the carboxysome for varying carboxysome permeabilities and a fixed ${\text{HCO}}_{3}^{-}$ transport rate in Figure 4. Figure 4A, shows that there is a broad range of *k*_*c*_ where the CCM has maximal efficacy. Figure 4 shows the distribution of inorganic carbon throughout the cell when the permeability is low (**B**), optimal (**C**), and high (**D**). At high permeability, the CO_2_ produced in the carboxysome rapidly leaks out of the carboxysome, and the CO_2_ concentration in the cytosol, shown in Figure 4D, is relatively high. When the carboxysome permeability decreases to near the optimal value, Figure 4C, the carboxysome traps CO_2_, and the cytosolic levels are lower, decreasing leakage out of the cell. This transition occurs when diffusion across the cell (and carboxysome) takes a shorter time than diffusion through the carboxysome shell; or the CO_2_ in the carboxysome is effectively partitioned from the CO_2_ in the cell.

If the carboxysome permeability is below optimal, diffusion of ${\text{HCO}}_{3}^{-}$ into the carboxysome cannot keep up with consumption from RuBisCO, Figure 4B. The existence of an optima requires RuBisCO consumption to be low enough that there is a *k*_*c*_ where the cytosol and carboxysome are partitioned, but ${\text{HCO}}_{3}^{-}$ diffusion in can keep up. When such an optima exists, the carboxysome permeability can improve the CO_2_ concentration in the carboxysome without any special selectivity between ${\text{HCO}}_{3}^{-}$ and CO_2_. The location and concentrating power of the optimal regime, is dependent on the size of the cell and the membrane permeabilities to CO_2_ and ${\text{HCO}}_{3}^{-}$.

## Discussion

### Are the fluxes and concentrations reasonable?

While we have solved our model to describe a vast parameter space it is instructive to compare the fluxes and concentrations we find within our optimal parameter space (the green region in Figure 2) to actual numbers. At low external inorganic carbon conditions, internal inorganic carbon pools due to CCM activity are regularly measured as high as C_i_ = 30 *mM*. The inorganic carbon is predominantly in the form of ${\text{HCO}}_{3}^{-}$, and measurements do not distinguish between the cytosol and carboxysome (Kaplan & Reinhold, 1999; Woodger et al., 2005; Sultemeyer et al., 1995; Price et al., 1998, 2008). In our model, we find that the cytosolic ${\text{HCO}}_{3}^{-}$ concentration is 30 mM when $j_{c}=0.6\frac{cm}{s}$, over a wide range of the carboxysome permeability (indicated as the dashed grey line in Figure 2). From Figure 4 we can also see that the cytosolic ${\text{HCO}}_{3}^{-}$ concentration is the dominate form of inorganic carbon in the cell at $j_{c}=0.6\frac{cm}{s}$. We examine the fate of the ${\text{HCO}}_{3}^{-}$ transported into the cell in terms of the ${\text{HCO}}_{3}^{-}$ leaking out, CO_2_ leaking out, CO_2_ fixation or carboxylation, and O_2_ fixation or oxygenation (Table 3).

**Table 3.** Fate of carbon brought into the cell for *j*_*c*_ = 0.6*cm/s* and *k*_*c*_ = 10^–3^*cm/s* **DOI:** http://dx.doi.org/10.7554/eLife.02043.013

	formula	$\left[\frac{picomoles}{\left(cell\;s\right)}\right]$	% of flux
${\text{HCO}}_{3}^{-}$ transport	*j*_*c*_*H*_*out*_	3.26 × 10^−4^	
${\text{HCO}}_{3}^{-}$ leakage	$k_{m}^{H}\left(H_{out}-H_{cytosol}(R_{b})\right)$	3.2 × 10^−4^	98.6%
CO_2_ leakage	$k_{m}^{C}\left(C_{out}-C_{cytosol}(R_{b})\right)$	4.5 × 10^−4^	1.4 %
carboxylation	$\frac{V_{\max}C}{C+K_{m}\left(1+\frac{O}{K_{O}}\right)}$	8.2 × 10^−8^	0.03 %
oxygenation	$\frac{V_{\max}^{O}C}{C+K_{m}^{O}\left(1+\frac{C}{K_{C}}\right)}$	6.7 × 10^−10^	2 × 10^−4^ %

For cells grown under low inorganic carbon conditions net ${\text{HCO}}_{3}^{-}$ fluxes (transport - leakage) are measured $10^{5}\frac{\text{pmol}}{\text{mgChls}}$, with CO_2_ net flux being slightly lower but the same order of magnitude (Whitehead et al., 2014; Badger et al., 1994). High external inorganic carbon conditions produce slightly higher net ${\text{HCO}}_{3}^{-}$ rates (Tchernov et al., 1997). Assuming chlorophyll per cell volume of around $10^{-11}\frac{\text{mgChl}}{\text{cell}}$ for cells of our size we can convert this into a flux of $10^{-6}\frac{pmol}{\mathrm{(c}ell\mathrm{s)}}$ (Whitehead et al., 2014; Keren et al., 2004, 2002). The net ${\text{HCO}}_{3}^{-}$ flux for our model cell is $6\times 10^{-6}\frac{pmol}{\mathrm{(c}ell\mathrm{s)}}$, so we are about an order of magnitude too high. If we choose a ${\text{HCO}}_{3}^{-}$ transport value one order of magnitude smaller, we will get net fluxes of the same order of magnitude as the measurements at the cost of slightly lower carboxylation rates and higher oxygenation rates (Table 4). This would also mean a lower internal ${\text{HCO}}_{3}^{-}$ pool. Alternatively, the same internal ${\text{HCO}}_{3}^{-}$ could be reached at a lower flux rate, if the external ${\text{HCO}}_{3}^{-}$ is higher. Since the majority of the ${\text{HCO}}_{3}^{-}$ transport is balanced by ${\text{HCO}}_{3}^{-}$ leakage, we can find the transport rate needed to sustain a particular amplification by the simple formula: $j_{c}=k_{m}^{H}(H_{out}-H_{cytosol}(R_{b}))/H_{out}$.

**Table 4.** Fate of carbon brought into the cell for *j*_*c*_ = 0.06 *cm/s* and *k*_*c*_ = 10^–3^*cm/s* **DOI:** http://dx.doi.org/10.7554/eLife.02043.014

	formula	$\left[\frac{picomoles}{\left(cell\;s\right)}\right]$	% of flux
${\text{HCO}}_{3}^{-}$ transport	*j*_*c*_*H*_*out*_	2.8 × 10^−5^	
${\text{HCO}}_{3}^{-}$ leakage	$k_{m}^{H}\left(H_{out}-H_{cytosol}(R_{b})\right)$	2.7 × 10^−5^	96.6 %
CO_2_ leakage	$k_{m}^{C}\left(C_{out}-C_{cytosol}(R_{b})\right)$	8.8 × 10^−7^	3.2 %
carboxylation	$\frac{V_{\max}C}{C+K_{m}\left(1+\frac{O}{K_{O}}\right)}$	5.4 × 10^−8^	0.2 %
oxygenation	$\frac{V_{\max}^{O}C}{C+K_{m}^{O}\left(1+\frac{C}{K_{C}}\right)}$	2.3 × 10^−9^	8 × 10^−3^ %

While we can compare the net fluxes, we have not found direct experimental evidence for the absolute ${\text{HCO}}_{3}^{-}$ uptake rate. To determine whether this ${\text{HCO}}_{3}^{-}$ transport rate is reasonable we perform a back of the envelope calculation. Our simulated cell has a flux of 2 × 10^8^ molecules/s. Assuming the rate of transport per transporter of $10^{3}\frac{\text{molecules}}{s}$ and our cell's surface area this requires about $10^{4}\frac{\text{transporters}}{\mu m^{2}}$. This is about an order of magnitude higher than the number of ATP synthase complexes on the thylakoid membrane in spinach, $700\;\frac{\text{complexes}}{\mu {\text{m}}^{2}}$ (Miller and Staehelin, 1979).

According to our calculation only around 1 % of the carbon transported into the cell is fixed into 3-phosophoglycerate. Even in this highly CO_2_ concentrating regime, 5 × 10^4^ 2-phosophoglycolate produced per second. Cyanobacteria have been shown to have multiple pathways for recycling 2-phosophoglycolate (Hackenberg et al., 2009). Our system fixes CO_2_ at a rate of 0.14 pg/hour. Given the volume of our cell, and the fact that between 115–300 fg/*μ*m^3^ of carbon are needed to produce a new cyanobacterial cell (Mahlmann et al., 2008) we need between 0.1 and 0.3 picograms of carbon per cell. At the higher flux rate (Table 3) this means that a cell could replicate every 7–21 hr and the lower flux rate (Table 4) allows replication every 11–35 hr. Both are consistent with the division times of cyanobacteria.

### Concentration profiles of CO_2_ and ${\text{HCO}}_{3}^{-}$ across the cell

At $j_{c}=0.6\frac{cm}{s}$, varying the carboxysome permeability changes how the available inorganic carbon is partitioned between the carboxysome and cytosol, thereby setting the carboxysomal CO_2_ concentration as shown in Figure 4. Strikingly, the ${\text{HCO}}_{3}^{-}$ concentration is constant across the cytosol. This is because the cell membranes have low permeability to ${\text{HCO}}_{3}^{-}$; thus, the rate of escape is slow and ${\text{HCO}}_{3}^{-}$ equilibrates across the cell. A consequence of this flat ${\text{HCO}}_{3}^{-}$ profile is that the carboxysome experiences the same ${\text{HCO}}_{3}^{-}$ concentration, independent of its position in the cell. This means the incoming inorganic carbon source for the carboxysome system is invariant with the position of the carboxysome in the cell.

In contrast, there is a gradient in CO_2_ concentration across the cell when the carboxysome permeability is at or above the optimum (Figure 4C,D). The cell membrane is more permeable to CO_2_. The gradient means that the CO_2_ leakage out of the cell affects the CO_2_ leakage out of the carboxysome. Moving the carboxysome close to the cell membrane increases the leakage rate of CO_2_ out of the carboxysome. Notably, in *S. elongatus* the carboxysomes are located along the center line of the cell, away from the cell membranes (Savage et al., 2010). The spatial profiles of ${\text{HCO}}_{3}^{-}$ and CO_2_ give no hint as to why the carboxysomes are spaced apart from one another. Since the gradient in ${\text{HCO}}_{3}^{-}$ is flat, there is no competition between the carboxysomes for ${\text{HCO}}_{3}^{-}$ (the main incoming source of inorganic carbon). In fact, since the local concentration of CO_2_ is higher near a carboxysome, nearby carboxysomes would ‘feed’ each other CO_2_. As has been shown, such clumping would reduce the probability of distributing carboxysomes equitably to daughter cells, possibly counteracting any benefit (Savage et al., 2010).

The concentration across the carboxysome is basically constant, because the carboxysome is so small that diffusion across it takes very little time. A consequence of this is that the organization of the reactions in the carboxysome does not effect the CO_2_ concentration in the carboxysome (Figure 3—figure supplement 1). Therefore, the localization of the carbonic anhydrase to the inner carboxysome shell seems to have no effect on the CCM. It has been suggested that diffusion in the carboxysome should be slower, since the carboxysome is packed with RuBisCO. One proposed consequence of slower diffusion in the carboxysome is that it could trap CO_2_, making a low carboxysome permeability unnecessary. We have tested this hypothesis (Figure 2—figure supplement 1), and find that assuming the diffusion constant one would expect for small molecules in a 60% sucrose solution ($D_{c}=10^{-7}\frac{cm^{2}}{s}$), does reduce the optimal carboxysome permeability. However, for any carboxysome permeability a higher ${\text{HCO}}_{3}^{-}$ transport rate is needed to achieve the same carboxsomal CO_2_ concentration. So if the diffusion is indeed slower in the carboxysome it does not aid the CCM. Even at this slower diffusion, the CO_2_ concentration across the carboxysome is flat.

### Benefit of CO_2_ to ${\text{HCO}}_{3}^{-}$ conversion: facilitated uptake or scavenging of CO_2_

We investigate the effect of CO_2_ to ${\text{HCO}}_{3}^{-}$ conversion at the thylakoid and cell membranes (combined in the model). Increasing conversion, *α* > 0, can facilitate uptake of CO_2_ from outside the cell and scavenge CO_2_ escaped from the carboxysome. Facilitated uptake results in saturating both carbonic anhydrase and RuBisCO at a lower level of ${\text{HCO}}_{3}^{-}$ transport. Scavenging broadens the range of carboxysome permeability which will effectively separate the inorganic carbon pools in the carboxysome and outside. Scavenging decreases the concentration of CO_2_ in the cytosol, so a more permeable carboxysome can still result in a low leakage rate of inorganic carbon out of the cell (more of the inorganic carbon in the cytosol is in the form of ${\text{HCO}}_{3}^{-}$ which leaks out less readily). However, neither of these effects is particularly strong in our current range of reaction rates, and cell membrane permeability (Figure 2—figure supplement 2).

**Figure 5.**
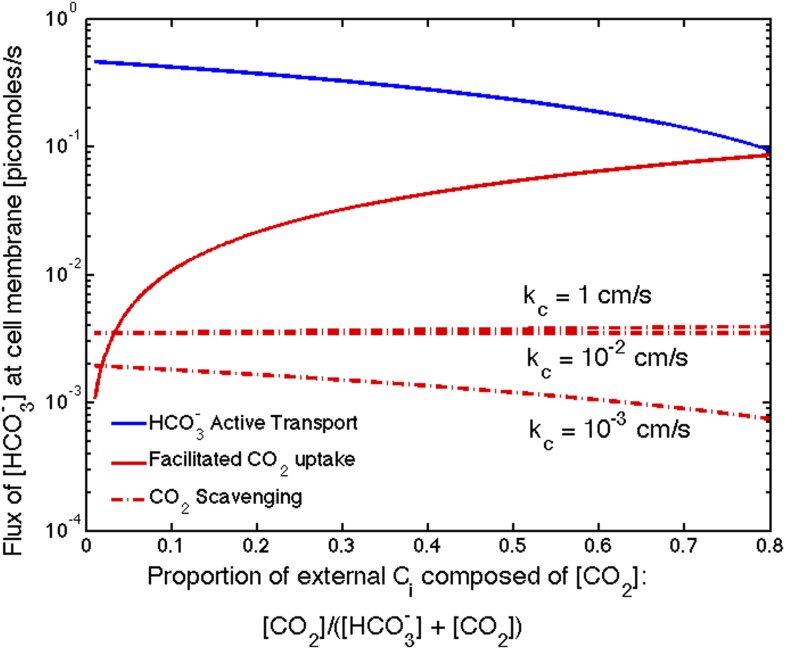
Size of the ${\text{HCO}}_{3}^{-}$ flux in one cell from varying sources, as the proportion of ${\text{CO}}_{2}$ to ${\text{HCO}}_{3}^{-}$ outside the cell changes changes. We show results for three carboxysome permeabilities, $k_{c}$, and only the scavenging is effected. Total external inorganic carbon is 15μM, $j_{c}=1\frac{cm}{s}$ and $\frac{\alpha }{K_{\alpha }}=1\frac{cm}{s}$. Scavenging is negligibly small for all values of $k_{c}$ shown. Unless there is very little ${\text{HCO}}_{3}^{-}$ in the environment, ${\text{HCO}}_{3}^{-}$ transport seems to be more efficient than ${\text{CO}}_{2}$ facilitated uptake. **DOI:** http://dx.doi.org/10.7554/eLife.02043.015

The relative effect of these two mechanisms depends on the external CO_2_ and ${\text{HCO}}_{3}^{-}$ concentrations. In saltwater environments the pH is near 8 and ${\text{HCO}}_{3}^{-}$ is the predominant inorganic carbon source. While external pH is not explicitly treated in our model, we can account for changes to pH through the external inorganic carbon concentration. To be consistent with oceanic environment, thus far we have shown results for low external inorganic carbon concentrations of [CO_2_] = 0.1 *μM* and [${\text{HCO}}_{3}^{-}$] = 14.9 *μM*. The effect of facilitated uptake, under these assumptions, is very small. In freshwater or under conditions of ocean acidification, where the pH could fall to 6 or lower, there can be a much larger proportion of CO_2_ (>50%). Figure 5 shows the absolute contribution of ${\text{HCO}}_{3}^{-}$ transport, facilitated CO_2_ uptake, and CO_2_ scavenging for varying proportions of external CO_2_. Even though we assume the same velocity of facilitated uptake and ${\text{HCO}}_{3}^{-}$ transport ($j_{c}=\frac{\alpha }{K_{\alpha }}=1$), facilitated uptake contributes less because it is limited by CO_2_ diffusion across the membrane. At the same rates of transport the facilitated uptake mechanism only contributes more than active ${\text{HCO}}_{3}^{-}$ if the CO_2_ concentration is greater than 80% of external inorganic carbon. This is consistent with observations that oceanic cyanobacteria such as Prochlorococcus only seem to possess gene homologs for ${\text{HCO}}_{3}^{-}$ transport systems, while other freshwater and estuarine cyanobacteria have gene homologs for both constitutive (NDH-1_4_) and inducible (NDH-1_3_) CO_2_ uptake systems as well as inducible ${\text{HCO}}_{3}^{-}$ transport systems (BicA, SbtA, and BCT1) (Price, 2011).

Scavenging is negligibly small for all values of ${\text{k}}_{\text{c}}$ shown. There is very little ${\text{CO}}_{2}$ in the cytosol, so there is very little ${\text{CO}}_{2}$ to scavenge, Figure 5. The effect of scavenging is dependent on the cell membrane permeability to ${\text{CO}}_{2}$ and ${\text{HCO}}_{3}^{-}$.

Given that scavenging has no obvious affect on ${\text{HCO}}_{3}^{-}$ concentrations, it is reasonable to wonder why this mechanism exists at all. One might assume that scavenging prevents leakage, but if the energy to bring a ‘new’ ${\text{HCO}}_{3}^{-}$ molecule from outside the cell is the same as the energy required to save an ‘old’ CO_2_ molecule from leaking out, there is no obvious advantage of preventing the leakage. It is possible that since the scavenging mechanism is associated with the electron transport chain of the light reactions of photosynthesis scavenging can be ramped up more easily when there is excess light energy. If this were the case, a comparison of $j_{c}=1\frac{cm}{s}$ and $\frac{\alpha }{k_{\alpha }}=1\frac{cm}{s}$ is deceiving and $\frac{\alpha }{K_{\alpha }}$ could be much larger. Indeed it has been suggested that the cell uses this mechanism as a way to dissipate excess light energy (Tchernov et al., 1997, 2003).

### Cellular organization

**Figure 6.**
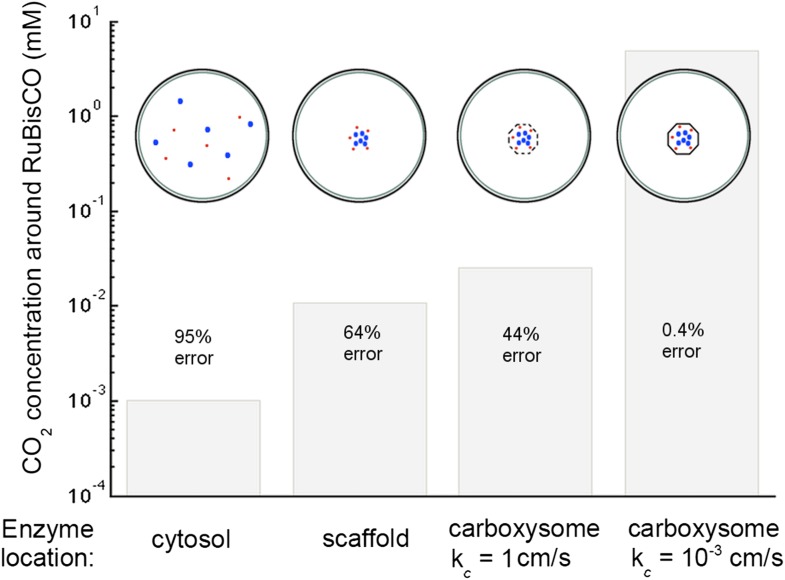
Concentration of CO_2_ achieved through various cellular organizations of enzymes, where we have selected the ${\text{HCO}}_{3}^{-}$ transport level such that the ${\text{HCO}}_{3}^{-}$ concentration in the cytosol is 30 *mM*. O_2_ concentration is 260 *μM*. The oxygenation error rates, as a percent of total RuBisCO reactions are indicated on the concentration bars. The cellular organizations investigated are RuBisCO and carbonic anhydrase distributed throughout the entire cytosol, co-localizing RuBisCO and carbonic anhydrase on a scaffold at the center of the cell without a carboxysome shell, RuBisCO and carbonic anhydrase encapsulated in a carboxysome with high permeability at the center of the cell, and RuBisCO and carbonic anhydrase encapsulated in a carboxysome with optimal permeability at the center of the cell. **DOI:** http://dx.doi.org/10.7554/eLife.02043.004

The most striking aspect of the CCM is the way that spatial organization is used to increase the efficacy of the reactions. Figure 6 compares the effect of different enzymatic reaction organizations. Concentrating carbonic anhydrase and RuBisCO to a small region in the center of the cell, on a scaffold for example, leads to an order of magnitude increase in the concentration of CO_2_. Localizing the carbonic anhydrase to a small volume concentrates it, increasing the maximum reaction rate per volume, *V*_*ca*_ and *V*_*ba*_. A larger *V*_*ba*_ increases the ${\text{HCO}}_{3}^{-}$ concentration at which carbonic anhydrase is saturated allowing the mechanism to take advantage of a larger ${\text{HCO}}_{3}^{-}$ flux, *j*_*c*_. A small increase can be gained from encapsulating the enzymes in a permeable carboxysome shell and another order of magnitude is gained at the optimal permeability. At optimal carboxysome permeability, the CO_2_ is effectively partitioned into the carboxysome and conversion can act only as facilitated uptake as shown in Figure 5.

Another advantage of localizing the enzymes in a small region at the center of the cell is separating carbonic anhydrase from the *α* (CO_2_ to ${\text{HCO}}_{3}^{-}$) conversion mechanism, preventing a futile cycle. The futile cycle is most detrimental when the enzymes are distributed through out the cytosol, and increases the oxygenation error rate (data not shown). Concentrating the enzymes away from the cell and thylakoid membranes, where conversion happens, removes this effect. On a scaffold the oxygenation rate is almost exactly the same with and without the *α* conversion mechanism. This is consistent with the previously shown detrimental effect of having active carbonic anhydrase free within the cytosol (Price & Badger, 1989). It would be impossible to keep the cytosol completely free from carbonic anhydrase enzyme, so there must be a way of activating it within the carboxysome only. Carbonic anhydrase is inactivated under reducing conditions (Peña et al., 2010). Recently it was shown that carboxysomes oxidize after assembly, providing a way to keep carbonic anhydrase inactive until fully enclosed in a carboxysome (Chen et al., 2013).

### Effects of pH

Cyanobacteria must regulate pH as almost all biochemical reactions are pH sensitive. We do not attempt to model this regulation or potential pH variation within the cell, however pH may be included implicitly in a couple ways. We have already explored the effect of varying external pH, and the effects of pH on carbonic anhydrase. Cytosolic pH would have little direct effect on the CO_2_ and ${\text{HCO}}_{3}^{-}$ levels since the non-enzymatic interconversion is very slow as previously discussed. The effect of internal pH could also be explored by varying the reaction rate of RuBisCO, which is pH sensitive. Varying the reaction rate of RuBisCO greatly could change the range where a non-specific carboxysome permeability can increase the concentration of CO_2_ in the carboxysome. It would be unexpected that the RuBisCO rate be much faster than we assume, as we have assumed a rate on the high end. A lower RuBisCO rate would increase the range of effective carboxysome permeabilities. As previously mentioned the CO_2_ facilitated uptake mechanism functions by creating local alkaline pockets. Diffusion of hydrogen ions across the cell would be very fast, so such pockets would require a massive reduction from the light reactions to maintain local alkalinity. Whether such pH gradients are possible, is certainly a subject of future interest.

### Conclusions

We have described and analyzed a model for the CO_2_ concentrating mechanism in cyanobacteria. There exists a broad range of ${\text{HCO}}_{3}^{-}$ transport and carboxysome permeability values which result in effective CO_2_ concentration in the carboxysome. This effective concentration parameter space is defined by CO_2_ levels high enough to saturate RuBisCO and produce a favorable ratio of carboxylation to oxygenation reactions, but not so high as to saturate carbonic anhydrase (after which increasing ${\text{HCO}}_{3}^{-}$ transport will not increase the CO_2_ concentration). An optimal carboxysome permeability exists, where ${\text{HCO}}_{3}^{-}$ diffusion into the carboxysome is not substantially inhibited, but CO_2_ leakage is minimal. ${\text{HCO}}_{3}^{-}$ concentrations across the cell are flat and are predominately set by the transport rate in, and leakage out. We quantitatively compare the transport rates and concentrations we predict in our optimal parameter space, and find them to be in good agreement with experiment. We also comment on the effects of external pH on CO_2_ versus ${\text{HCO}}_{3}^{-}$ uptake mechanisms. Finally we describe the cumulative benefits of co-localization, encapsulation, and optimal carboxysome permeability on the CCM.

Further comparison of this model to experimental flux measurements, especially to determine the quantitative contributions of different transporters under different physiological conditions would be very interesting. Current solutions are for steady state at constant external concentration, but most gas exchange measurements, by necessity, measure the fluxes as the inorganic carbon is depleted in the media. The model could be modified to solve the time dependent problem with varying external inorganic carbon. As of yet the carboxysome permeability has not been measured directly, and it would be quite interesting to see how close it is to our ‘optimal’ prediction.

## Additional files

Supplementary file 1. Mathematical derivation appendix. Mathematical derivations of analytic solutions for a spherical cell with reactions organized in a variety of ways. We present analytic solutions for the concentration of CO_2_ and HCO_3_^−^ a carboxysome located at the center of the cell. We derive analytic solutions assuming a number of different cases for the enzymatic rates in the carboxysome: RuBisCO reaction rate negligible with carbonic anhydrase saturated and unsaturated, RuBisCO reaction rate non-negligible with carbonic anhydrase unsaturated. Additionally we derive analytic solutions for the enzymatic reactions throughout the cell and localized to a scaffold without a carboxysome shell. **DOI:** http://dx.doi.org/10.7554/eLife.02043.016
