# Correction: Systems analysis of the CO_2_ concentrating mechanism in cyanobacteria

**DOI:** 10.7554/eLife.06400

**Published:** 2015-03-26

**Authors:** Niall M Mangan, Michael P Brenner

Mangan NM, Brenner MP. 2014. Systems analysis of the CO_2_ concentrating mechanism in cyanobacteria. *eLife* **3**:e02043. doi: 10.7554/eLife.02043

Published 29 April 2014

We would like to issue the following corrections to our research article entitled, ‘Systems analysis of the CO_2_ concentrating mechanism in cyanobacteria.’ The code used to generate the plots in the paper, mistakenly used 6.022 × 10^22^ instead of 6.022 × 10^23^ for Avogadro's number. This correction changes the quantitative numbers in the original paper but none of the conclusions are affected. Here are the corrected tables, figures, and text that changed. We thank Avi Flamholz for catching this error.

The article has been corrected accordingly. The original version published on 29 April 2014 is provided as Supplementary file 1 (file held on figshare under doi: 10.6084/m9.figshare.1352030).

## Table 2. Enzymatic rates

We recalculated the enzymatic rates for carbonic anhydrase and RuBisCO. The values for V_max_ and K_1/2_ listed in the previous version of Table 2 used the incorrect 6.022 × 10^22^ value for Avogadro's number. All plots in the original paper were for those values. All new plots are for the values listed in the updated table, using Avogadro's number 6.022 × 10^23^. The V_max_ values are all about an order of magnitude lower than previously calculated.

**Table:** 

Enzyme reaction	active sites	*k*_*cat*_ $\left[\frac{1}{s}\right]$	*V*_*max*_ in ‘cell’ $\left[\frac{\mu M}{s}\right]$	*V*_*max*_ in carboxysome $\left[\frac{\mu M}{s}\right]$	*K*_*1/2*_ [μM]
carbonic anhydrase hydration	80	8 × 10^4^	8.8 × 10^3^	1.5 × 10^7^	3.2 × 10^3^
carbonic anhydrase dehydration	80	4.6 × 10^4^	1.5 × 10^4^	8.8 × 10^6^	9.3 × 10^3^
RuBisCO carboxylation	2160	26	178	1.8 × 10^5^	270

## Figure 2. Phase space for ${\text{HCO}}_{3}^{-}$ transport and carboxysome permeability

The main effect of correcting Avagadro's number is that the peak defining optimal carboxysome permeability is lower and broader. The optimal carboxhysome permeability for a target carboxysomal CO_2_ concentration is found by looking at the leftmost value on a line of constant concentration on the permeability vs ${\text{HCO}}_{3}^{-}$ transport plot. This point represents the carboxysome permeability where the least amount of ${\text{HCO}}_{3}^{-}$ transport is needed to achieve a given carboxysomal CO_2_ concentration. For 99% carboxylation (cyan curve below) this value is now around *k*_*c*_ = 10^−3^ cm/s (previously *k*_*c*_ = 6 × 10^−3^ cm/s). Carbonic anhydrase also saturates at a lower ${\text{HCO}}_{3}^{-}$ transport value.

**Figure 2.**
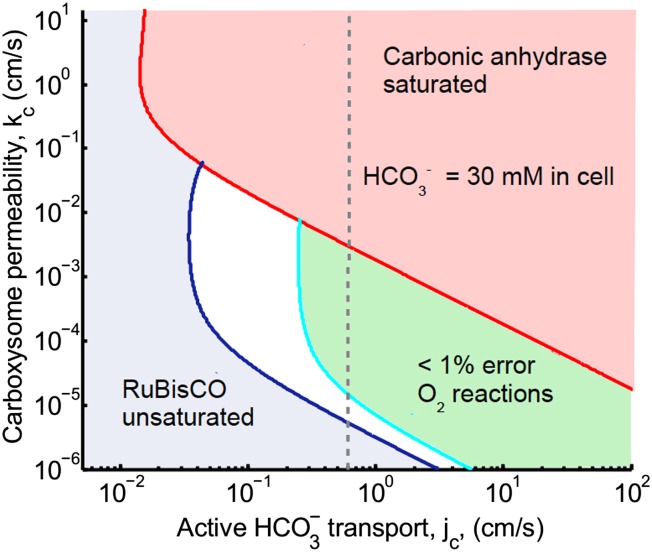
Plotted are the parameter values at which the CO_2_ concentration reaches some critical value. The leftmost line (dark blue) indicates for what values of *j*_*c*_ and *k*_*c*_ the CO_2_ concentration in the carboxysome would half-saturate RuBisCO (*K*_*m*_). The middle line (light blue) indicates the parameter values which would result in a CO_2_ concentration where 99% of all RuBisCO reactions are carboxylation reactions and only 1% are oxygenation reactions when O_2_ concentration is 260 *μ*M. To the left of the line the error is greater and to the right it is smaller. The right most (red) line indicates the parameter values which result in carbonic anhydrase saturating. Here the rate of conversion from CO_2_ to ${\text{HCO}}_{3}^{-}$, *α* = 0, so there is no CO_2_ scavenging or facilitated uptake of CO_2_. The dotted line (grey) shows the *k*_*c*_ and *j*_*c*_ values, where the ${\text{HCO}}_{3}^{-}$ concentration in the cytosol is 30 mM. The ${\text{HCO}}_{3}^{-}$ concentration in the cytosol does not vary appreciably with *k*_*c*_ in this parameter regime, and reaches 30 mM at $j_{c}\approx 0.6\;cm/s$. All other parameters, such as reaction rates are held fixed and the values can be found in the Tables 1 and 2.

## Figure 2—figure supplement 1. Effect of decreased diffusion in the carboxysome

With the correction, decreasing the diffusion constant in the carboxysome has much less of an effect on the phase space plot. For optimal or larger carboxysome permeability, there is very little effect (*k*_c_ ≥ 10^−3^ cm/s).

**Figure 2—figure supplement 1.**
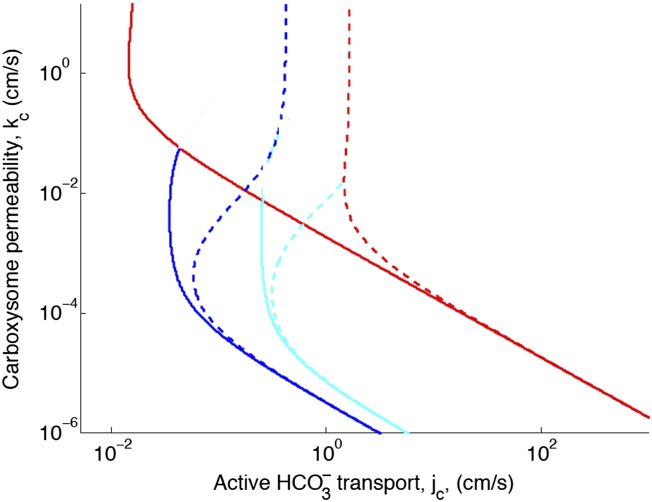
Phase space for ${\text{HCO}}_{3}^{-}$ transport and carboxysome permeability for varying diffusion in the carboxysome. Solid lines show lines of constant CO_2_ concentration in the carboxysome for $D_{c}=1\times 10^{-5}\;\frac{{\text{cm}}^{2}}{\text{s}}$, or the diffusion constant of small molecule in water. Dashed lines show the same lines of constant CO_2_ concentration, bur for $D_{c}=1\times 10^{-7}\;\frac{{\text{cm}}^{2}}{\text{s}}$, or the diffusion constant of a small molecule in a 60% sucrose solution.

## Figure 2—figure supplement 2. Effect of CO_2_ scavenging or facilitated uptake

The correction reduces the effect of facilitated uptake. At the optimal permeability it has nearly no effect (difference between dashed and solid lines near *k*_c_ = 10^−3^ cm/s).

**Figure 2—figure supplement 2.**
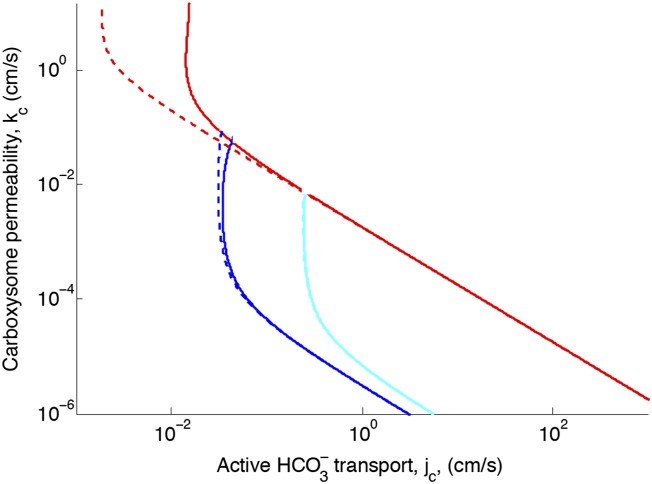
Effect of CO2 scavenging or facilitated uptake on phase space for ${\text{HCO}}_{3}^{-}$ transport, *j*_*c*_, and carboxysome permeability, *k*_*c*_. Plotted are the parameter values at which CO_2_ concentration reaches some critical value. The leftmost line (dark blue) indicates for what values of *j*_*c*_ and *k*_*c*_ the CO_2_ concentration in the carboxysome would saturate RuBisCO. The middle line (light blue) indicates the parameter values which would result in a CO_2_ concentration where 99% of all RuBisCO reactions are carboxylation reactions and only 1% are oxygenation reactions when O2 concentration is 260 *μ*M. The rightmost (red) line indicates the parameter values which result in carbonic anhydrase saturating. Here *α* = 0 cm/s (solid lines) and *α* = 1 cm/s (dashed line), showing the effect of CO2 scavenging or facilitated uptake on the phase space. All other parameters, such as reaction rates are held fixed and the values can be found Table 1.

## Figure 3. CO_2_ concentration in the carboxysome as function of ${\text{HCO}}_{3}^{-}$ transport: comparison of numeric and analytic solutions

We plot Figure 3 assuming the new optimal carboxysome permeability of *k*_*c*_ = 10^−3^ cm/s (previously *k*_*c*_ = 6 × 10^−3^ cm/s). There is very little change from the previous Figure 3, with the updated carboxysome permeability. Had we plotted at the same *k*_*c*_ = 6 × 10^−3^ cm/s, the CO_2_ would have been lower, because the system would not be at the optimal permeability.

**Figure 3.**
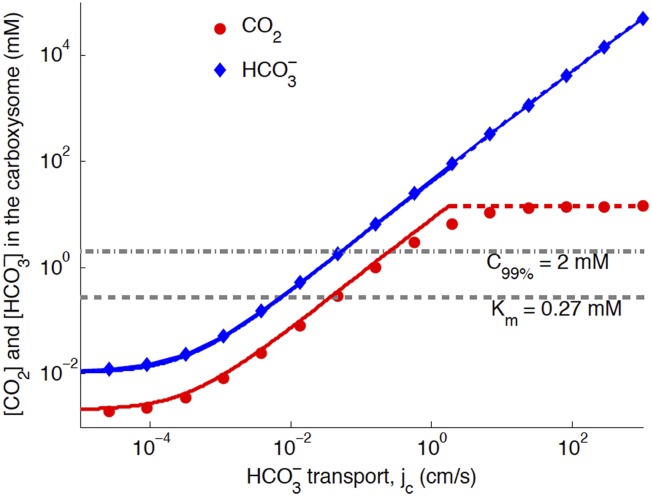
Numerical solution (diamonds and circles) and analytic solutions (carbonic anhydrase unsaturated, solid lines, and saturated, dashed lines) correspond well. ${\text{HCO}}_{3}^{-}$ transport is varied, and all other system parameters are held constant. The CO_2_ concentration above which RuBisCO is saturated is *K*_*m*_ (grey dashed line). The CO_2_ concentration where the oxygen reaction error rate will be 1% is C_99%_ (grey dash-dotted line). The transition between carbonic anhydrase being unsaturated and saturated happens where the two analytic solutions meet (where the dashed and solid red lines meet). Below a critical value of transport, $j_{c}\approx 10^{-3}\text{ cm}/\text{s}$ the level of transport is lower than the ${\text{HCO}}_{3}^{-}$ leaking through the cell membrane. For these simulations and solutions the carboxysome permeability was *k*_c_ = 10^−3^ cm/s (in previous version *k*_*c*_ = 6 × 10^−3^ cm/s was used).

## Figure 3—figure supplement 1. No effect of localizing carbonic anhydrase to the shell of the carboxysome

The correction did not at all effect the conclusion that the organization of carbonic anhydrase within the carboxysome has no effect on the resulting concentrations. Consistent with updated Figure 2—figure supplement 1, the effect of decreasing the diffusion in carboxysome is much less prominent than in the original manuscript.

**Figure 3—figure supplement 1.**
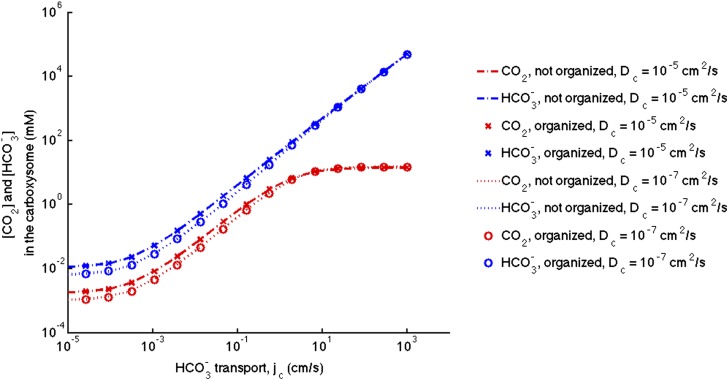
No effect of localizing carbonic anhydrase to the shell of the carboxysome. We assume the same amount of carbonic anhydrase and RuBisCO activity for each simulation and compare the case with the enzymes evenly distributed throughout the carboxysome to the case where the carbonic anhydrase is localized to the inner carboxysome shell. The (-.-) lines are for no organization and (x) for organized with *D*_*c*_ = 10^−5^ cm^2^/s. The (…) lines are for no organization and (o) are for organized with *D*_*c*_ = 10^−5^ cm^2^/s.

## Figure 4. CO_2_ concentration in the carboxysome with varying carboxysome permeability

The correction does not effect the conclusion that an optimal carboxysome permeability exists, or what causes it, but it changes its absolute value. The correction has two main effects: (1) it decreases the optimal carboxysome permeability (seen as a shift in the peak) by a factor of 6, and (2) for carboxysome permeabilities higher than optimal (*k*_c_ > 0.1 cm/s) the CO_2_ concentration is an order of magnitude lower. Therefore the optimal carboxysome permeability peak is at a lower *k*_*c*_ value and more prominent.

**Figure 4.**
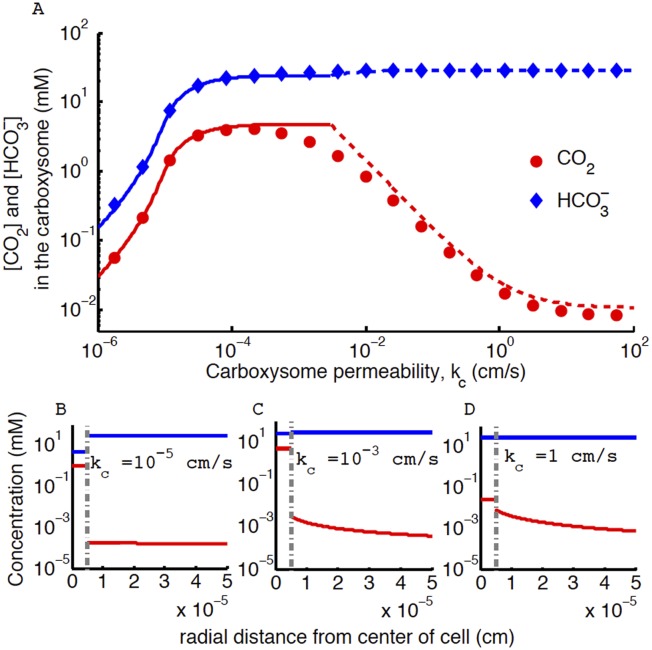
Concentration of CO_2_ in the carboxysome with varying carboxysome permeability. (**A**) Numerical solution (diamonds and circles) and analytic solutions (carbonic anhydrase unsaturated, solid lines, and saturated, dashed lines) correspond well. On all plots CO_2_ (red circle) $<{\text{HCO}}_{3}^{-}$ (blue diamond). Concentration in the cell along the radius, r, with carboxysome permeability *k*_*c*_ = 10^−5^ cm/s (**B**), *k*_*c*_ = 10^−3^ cm/s (**C**), *k*_*c*_ = 1 cm/s (**D**). Grey dotted lines in (**B**), (**C**), (**D**) indicate location of the carboxysome shell boundary. The transition from low CO_2_ at high permeability (**D**) to maximum CO2 concentration at optimal permeability (**C**) occurs at $k_{c}=\frac{D}{R_{C}}=2\;cm/s$. At low carboxysome permeability (**B**) ${\text{HCO}}_{3}^{-}$ diffusion into the carboxysome is slower than consumption. For all subplots the rate of CO_2_ to ${\text{HCO}}_{3}^{-}$ conversion at the cell membrane, *α* = 0 *cm/s* and ${\text{HCO}}_{3}^{-}$ uptake, *j*_*c*_ *=* 0.6 *cm/s*. Qualitative results remain the same with varying *j*_*c*_, increasing *α* will increase the gradient of CO_2_ across the cell as CO_2_ is converted to ${\text{HCO}}_{3}^{-}$ at the cell membrane.

## Table 3. Fate of carbon brought into the cell for *j*_*c*_ *=* 0.6 *cm/s* and *k*_*c*_ = 1 × 10^−3^ *cm/s*

We updated the flux to the value at which cytosolic ${\text{HCO}}_{3}^{-}$ is 30 mM and carboxysome permeability is optimal. Previously they were *j*_*c*_ *=* 0.7 *cm/s* and *k*_*c*_ *=* 6 × 10^−3^ *cm/s.*

**Table 3.** Fate of carbon brought into the cell for *j*_*c*_ *=* 0.6 *cm/s* and *k*_*c*_ *=* 1 × 10^−3^ *cm/s*

	Formula	$\left[\frac{picomoles}{\left(cell\;s\right)}\right]$	% Of flux
${\text{HCO}}_{3}^{-}$ transport	*j*_*c*_*H*_*out*_	3.26 × 10^−4^	
${\text{HCO}}_{3}^{-}$ leakage	$k_{m}^{H}\left(H_{out}-H_{cytosol}\left(R_{b}\right)\right)$	3.2 × 10^−4^	98.6%
CO_2_ leakage	$k_{m}^{C}\left(C_{out}-C_{cytosol}\left(R_{b}\right)\right)$	4.5 × 10^−6^	1.4%
carboxylation	$\frac{V_{max}C}{C+K_{m}\left(1+\frac{O}{K_{O}}\right)}$	8.2 × 10^−8^	0.03%
oxygenation	$\frac{V_{max}^{O}C}{C+K_{m}^{O}\left(1+\frac{C}{K_{C}}\right)}$	6.7 × 10^−10^	2 × 10^−4^ %

## Table 4. Fate of carbon brought into the cell for *j*_*c*_ *=* 0.06 *cm/s* and *k*_*c*_ *=* 1 *×* 10^−3^ *cm/s*

**Table 4.** Fate of carbon brought into the cell for *j*_*c*_ *=* 0.06 *cm/s* and *k*_*c*_ *=* 1 *×* 10^−3^ *cm/s*

	Formula	$\left[\frac{picomoles}{\left(cell\;s\right)}\right]$	% Of flux
${\text{HCO}}_{3}^{-}$ transport	*j*_*c*_*H*_*out*_	2.8 × 10^−5^	
${\text{HCO}}_{3}^{-}$ leakage	$k_{m}^{H}\left(H_{out}-H_{cytosol}\left(R_{b}\right)\right)$	2.7 × 10^−5^	96.6%
CO_2_ leakage	$k_{m}^{C}\left(C_{out}-C_{cytosol}\left(R_{b}\right)\right)$	8.8 × 10^−7^	3.2%
carboxylation	$\frac{V_{max}C}{C+K_{m}\left(1+\frac{O}{K_{O}}\right)}$	5.4 × 10^−8^	0.2%
oxygenation	$\frac{V_{max}^{O}C}{C+K_{m}^{O}\left(1+\frac{C}{K_{C}}\right)}$	2.3 × 10^−9^	8 × 10^−3^%

## Discussion of fluxes and resulting 2-phosphoglycolate, and 3-phosphoglycerate production

The discussion of the number of transporters needed for this magnitude of ${\text{HCO}}_{3}^{-}$ influx increases by an order of magnitude from $10^{3}\;\frac{\text{transporters}}{\mu {\text{m}}^{2}}$ to $10^{4}\;\frac{\text{transporters}}{\mu {\text{m}}^{2}}$. While the change in uptake is less than a factor of two different, the conversion from picomoles/s to molecules/s for a cell used the incorrect value of Avogadro's number. So this is now about an order of magnitude larger than the number of ATP synthase complexes on the thylakoid membrane of spinach.

The numbers for net flux of ${\text{HCO}}_{3}-$ change slightly. For *j*_*c*_ *=* 0.6 *cm/s* the net flux is $6\times 10^{-6}\frac{\text{pmol}}{\text{cell s}}$ compared to the previous value of $10^{-5}\frac{\text{pmol}}{\text{cell s}}$ and for *j*_*c*_ *=* 0.06 *cm/s* the net flux is $9\times 10^{-7}\;\frac{\text{pmol}}{\text{cell s}}$ compared with the previous value of $1\times 10^{-6}\;\frac{\text{pmol}}{\text{cell s}}$. So the conclusion that measured net fluxes of $10^{-6}\frac{\text{pmol}}{\text{cell s}}$ compares best with the lower ${\text{HCO}}_{3}^{-}$ flux still holds.

Since Avagadro's number effects the rate of carboxylation and oxygenation, the recalculated values for 2-phosphoglycolate and 3-phosphoglycerate production are lower. This exacerbates the large amount of energy that appears to go into CO_2_ concentration compared to the actual fixation. Now around 0.03% of concentrated inorganic carbon is fixed into 3-phosphoglycerate. Over 99.9% is lost to leakage either in the form of CO_2_ or ${\text{HCO}}_{3}^{-}$ (previously these values were calculated to be 99%). The higher ${\text{HCO}}_{3}^{-}$ flux assumption (Table 3) predicts that only 217 2-phosphoglycolate are produced a second, and the lower ${\text{HCO}}_{3}^{-}$ flux assumption (Table 4) predicts that 1.4 × 10^3^ 2-phosphoglycolate are produced a second. So the higher flux allows the production of many fewer 2-phosphoglycolate than previously estimated. Recalculating the time to fix the necessary carbon to replicate a cell results in 7–21 hr for the higher flux rate (Table 3) and 11–35 hr for the lower flux rate (Table 4). Both are consistant with the division times of cyanobacteria, but the higher flux rate is a better match.

In conclusion, the correction further highlights the puzzle of how much energy cyanobacteria must use ${\text{HCO}}_{3}^{-}$ to achieve a level of CO_2_ which creates adequate fixation rates. Either cyanobacteria use a much larger amount of energy to achieve this internal concentration than to fix each CO_2_, the permeability of the cell must be much lower for ${\text{HCO}}_{3}^{-}$ than assumed here, or there is another mechanism not covered in this model.

## Figure 5. Flux of ${\text{HCO}}_{3}^{-}$ from varying sources as the proportion of CO_2_ to ${\text{HCO}}_{3}^{-}$ outside the cell changes

The correction decreases the effect of CO_2_ scavenging at high permeabilities (*k*_*c*_ = 1 cm/s and 10^−2^ cm/s). It now appears to be negligible compared to ${\text{HOC}}_{3}^{-}$ uptake and CO_2_ uptake for all permeabilities. Only when CO_2_ concentration is a very small percentage of the external carbon, does the scavenging contribute more than the CO_2_ uptake, and both are far less than ${\text{HCO}}_{3}^{-}$ uptake.

**Figure 5.**
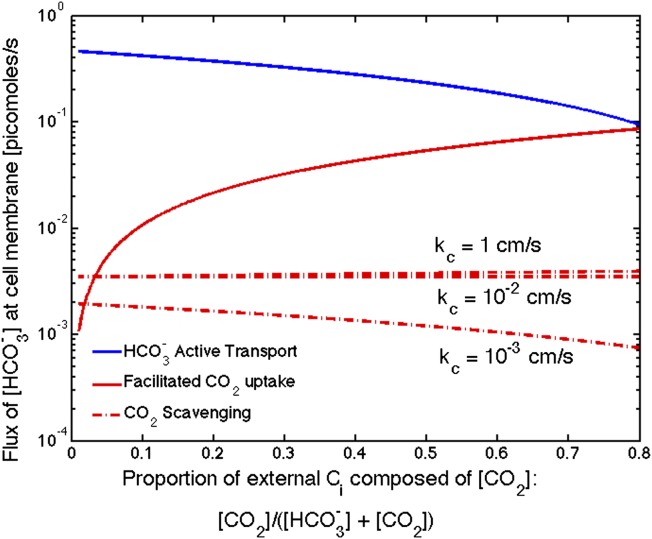
Size of the ${\text{HCO}}_{3}^{-}$ flux in one cell from varying sources, as the proportion of CO_2_ to ${\text{HCO}}_{3}^{-}$ outside the cell changes changes. We show results for three carboxysome permeabilities, *k*_*c*_, and only the scavenging is effected. Total external inorganic carbon is 15 *μ*M, *j*_*c*_ = 1 cm/s and $\frac{\alpha }{K_{\alpha }}=1\text{ cm}/\text{s}$. Scavenging is negligibly small for all values of *k*_*c*_ shown. Unless there is very little ${\text{HCO}}_{3}^{-}$ in the environment, ${\text{HCO}}_{3}^{-}$ transport seems to be more efficient than CO_2_ facilitated uptake.

## Figure 6. CO_2_ concentration with various organizations of enzymes in the cell

With the corrected Avagadro's number the CO_2_ concentration is significantly lower in all but the optimal permeability case, making the error rate drastically higher. The effect of optimal permeability on CO_2_ concentration is, therefore, much larger.

**Figure 6.**
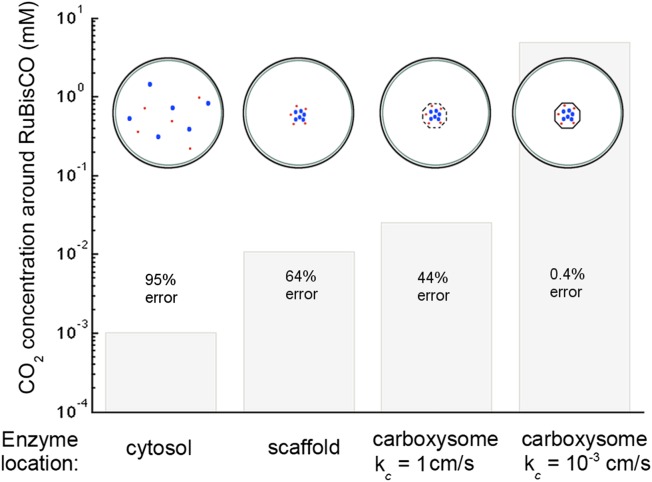
Concentration of CO_2_ achieved through various cellular organizations of enzymes, where we have selected the ${\text{HCO}}_{3}^{-}$ transport level such that the ${\text{HCO}}_{3}^{-}$ concentration in the cytosol is 30 mM. O2 concentration is 260 *μ*M. The oxygenation error rates, as a percent of total RuBisCO reactions are indicated on the concentration bars. The cellular organizations investigated are RuBisCO and carbonic anhydrase distributed throughout the entire cytosol, co-localizing RuBisCO and carbonic anhydrase on a scaffold at the center of the cell without a carboxysome shell, RuBisCO and carbonic anhydrase encapsulated in a carboxysome with high permeability at the center of the cell, and RuBisCO and carbonic anhydrase encapsulated in a carboxysome with optimal permeability at the center of the cell.

## Corrections to text

Throughout the text the default ${\text{HCO}}_{3}^{-}$ flux has been changed from $j_{c}=0.7\frac{\text{cm}}{\text{s}}$ to $j_{c}=0.6\frac{\text{cm}}{\text{s}}$ and optimal carboxysome permeability from $k_{c}=6\times 10^{-3}\frac{\text{cm}}{\text{s}}$ to $k_{c}=10^{-3}\frac{\text{cm}}{\text{s}}$.

- In the discussion of fluxes and concentrations we previously had written:

‘Our simulated cell has a flux of 2 × 10^7^ molecules/s. Assuming the rate of transport per transporter of $10^{3}\frac{\text{molecules}}{\text{s}}$ and our cell’s surface area this requires about $10^{3}\frac{\text{transporters}}{\mu {\text{m}}^{2}}$. This is actually not that far off from the number of ATP synthase complexes on the thylakoid membrane in spinach, $700\frac{\text{complexes}}{\mu {\text{m}}^{2}}$ (Miller and Staehelin, 1979), although it is still quite high.’

This is now: ‘Our simulated cell has a flux of 2 × 10^8^molecules/s. Assuming the rate of transport per transporter of $10^{3}\frac{\text{molecules}}{\text{s}}$ and our cell’s surface area this requires about $10^{4}\frac{\text{transporters}}{\mu {\text{m}}^{2}}$. This is about an order of magnitude higher than the number of ATP synthase complexes on the thylakoid membrane in spinach, $700\frac{\text{complexes}}{\mu {\text{m}}^{2}}$ (Miller and Staehelin, 1979).’

- In the discussion of fluxes and concentrations we previously had written:

‘At the higher flux rate (Table 3) this means that a cell could replicate every 1–2 hr, so faster than cyanobacteria replicate. The lower flux rate (Table 4) would produce fix enough CO_2_ for the cell to replicate every 8 to 21 hr, which is similar to the division times of cyanobacteria.’

This is now: ‘At the higher flux rate (Table 3) this means that a cell could replicate every 7–21 hr and the lower flux rate (Table 4) allows replication every 11 to 35 hr. Both are consistent with the division times of cyanobacteria.’

- In the caption of Figure 5 we had written:

‘When the carboxysome permeability is larger than optimal, *k*_*c*_ = 1 cm/s, scavenging can contribute more than facilitated uptake at low external CO_2_ concentrations. However, when the carboxysome permeability at or below our geometric bound, *k*_*c*_ <= 0.02 *cm/s*, scavenging is negligibly small.’

This is now: ‘Scavenging is negligibly small for all values of *k*_*c*_ shown.’

- In the text of the Benefit of CO_2_ to ${\text{HCO}}_{3}^{-}$ conversion: facilitated uptake or scavenging of CO_2_ we had written: ‘Scavenging only contributes significantly to total incoming ${\text{HCO}}_{3}^{-}$ when the carboxysome permeability is higher than optimal, Figure 5, and does not contribute significantly below our calculated upper bound of $k_{c}<=0.02cm/s$. In these ranges for carboxysome permeability, there is very little CO2 leaking out of the carboxysome into the cytosol, so there is very little CO2 to scavenge, Figure 5.’

This is now: ‘Scavenging is negligibly small for all values of k_c_ shown. There is very little CO_2_ in the cytosol, so there is very little CO_2_ to scavenge, Figure 5.’

- There were also a few typos we have now corrected:
  1. In the Reaction diffusion model section we had: ‘RuBisCO also requires ribulose-1,5-bisphosphate, the substrate which CO_2_ reacts with to produce 3-phosphoglycolate.’ This has been corrected to ‘… CO_2_ reacts with to produce 3-phosphoglycerate.’
  2. in Table 1: ‘$k_{m}^{H}$ permeability of cell membrane to CO_2_’ has been corrected to: ‘$k_{m}^{H}$ permeability of cell membrane to ${\text{HCO}}_{3}^{-}$’.
  3. Table 2 header said *k*_*cut*_ which has been corrected to *k*_*cat*_.
  4. In the Varying ${\text{HCO}}_{3}^{-}$ transport saturates enzyme section we had:
    - The chemical equilibrium is $K_{eq}=H/C=\left(K_{ba}V_{ca}\right)\left(K_{ca}V_{ba}\right)\approx 5$, for pH around 7 (DeVoe and Kistiakowsky, 1961), so that ${\text{HCO}}_{3}^{-}$ > CO_2_ in the carboxysome.
    - The equation is now: $K_{eq}=H/C=\left(KbaVca\right)/\left(KcaVba\right)\approx 5$.
  5. In Figure 3—figure supplement 1 the units for *D*_*c*_ on both the plot and caption are cm/s, they should be *cm*^*2*^*/s*.

