# Supplementary material for: Systems analysis of the CO2 concentrating mechanism in cyanobacteria
Source: eLife. 2014 Apr 29;3:e02043. doi: 10.7554/eLife.02043 (PMC4027813; doi:10.7554/eLife.02043)
Supplement: Supplementary file 1. — Mathematical derivation appendix. Mathematical derivations of analytic solutions for a spherical cell with reactions organized in a variety of ways. We present analytic solutions for the concentration of CO2 and HCO3− a carboxysome located at the center of the cell. We derive analytic solutions assuming a number of different cases for the enzymatic rates in the carboxysome: RuBisCO reaction rate negligible with carbonic anhydrase saturated and unsaturated, RuBisCO reaction rate non-negligible with carbonic anhydrase unsaturated. Additionally we derive analytic solutions for the enzymatic reactions throughout the cell and localized to a scaffold without a carboxysome shell. DOI: http://dx.doi.org/10.7554/eLife.02043.016 [file elife02043s001.pdf]

Supplementary File 1: Analytic equations derevation

S1 Equations in the cytosol

The equations for diffusion of HCO_3^-, H, and CO_2, C, in the cytosol are

$$\partial_t C = D\nabla^2 C \tag{1}$$
$$\partial_t H = D\nabla^2 H. \tag{2}$$

Here D is the diffusion coefficient.

At steady state and in spherical coordinates the solutions to $\nabla^2 C = 0$ and $\nabla^2 H = 0$ are known; they have the form

$$C = \frac{A_3}{r} + A_4 \tag{3}$$
$$H = \frac{B_3}{r} + B_4 \tag{4}$$

where A_3, A_4, B_3, and B_4 are constants set by the boundary conditions.

The boundary condition at the cell membrane sets the gradient;

$$D\frac{\partial C}{\partial r} = -\frac{\alpha C_{cytosol}}{K_\alpha + C_{cytosol}} + k_m^C\left(C_{out} - C_{cytosol}\right) \tag{5}$$

$$D\frac{\partial H}{\partial r} = j_c H_{out} + \frac{\alpha C_{cytosol}}{K_\alpha + C_{cytosol}} + k_m^H\left(H_{out} - H_{cytosol}\right) \tag{6}$$

Here active transport of HCO_3^- is set by the transport velocity j_c. Conversion of CO_2 to HCO_3^- has maximum velocity α and half maximum concentration K_α. The permeability of the cell membrane to CO_2 and HCO_3^- are set by escape velocities k_m^C and k_m^H. We will assume the reactions are unsaturated, so $\frac{\alpha C_{cytosol}}{K_\alpha + C_{cytosol}} \approx \frac{\alpha}{K_\alpha} C_{cytosol}$.

Similarly the gradient at the carboxysome shell sets the linking boundary condition between the concentrations inside the carboxysome and in the cytosol.

$$D\frac{\partial C}{\partial r} = k_c(C_{cytosol} - C_{carboxysome}) \tag{7}$$
$$D\frac{\partial H}{\partial r} = k_c(H_{cytosol} - H_{carboxysome}). \tag{8}$$

Here the velocity of transport across the carboxysome shell is k_c.

S1.1 Solution in cytosol

Using equation 1.3 in boundary condition 1.7 and equation 1.4 in boundary condition 1.7, we obtain:

$$A_4 = C_{carboxysome} - A_3\left(\frac{D}{k_c R_c^2} + \frac{1}{R_C}\right) \tag{9}$$
$$B_4 = H_{carboxysome} - B_3\left(\frac{D}{k_c R_c^2} + \frac{1}{R_C}\right) \tag{10}$$

so

$$C = A_3\left(\frac{1}{r} - \frac{D}{k_c R_c^2} - \frac{1}{R_C}\right) + C_{carboxysome} \tag{11}$$

$$H = B_3\left(\frac{1}{r} - \frac{D}{k_c R_c^2} - \frac{1}{R_C}\right) + H_{carboxysome} \tag{12}$$

Then using 1.11 in 1.5 and 1.12 in 1.6 we find;

$$A_3 = \frac{(\frac{\alpha}{K_\alpha} + k_m^C)C_{carboxysome} - k_m^C C_{out}}{(\frac{\alpha}{K_\alpha} + k_m^C)G + \frac{D}{R_b^2}} \tag{13}$$

$$B_3 = \frac{(k_m^H H_{carboxysome} - (j_c + k_m^H)H_{out} - \frac{\alpha}{K_\alpha}C_{cytosol}(r = R_b))}{k_m^H G + \frac{D}{R_b^2}} \tag{14}$$

We have grouped the following parameters:

$$G = \left(\frac{D}{R_c^2 k_c} + \frac{1}{R_c} - \frac{1}{R_b}\right) \tag{15}$$

Using our values for these constants we find the equations for CO_2 and HCO_3^- in the cytosol;

$$C_{cytosol} = \frac{k_m^C C_{out} - (\frac{\alpha}{K_\alpha} + k_m^C)C_{carboxysome}}{(\frac{\alpha}{K_\alpha} + k_m^C)G + \frac{D}{R_b^2}}\left(\frac{D}{k_c R_c^2} + \frac{1}{R_C} - \frac{1}{r}\right) + C_{carboxysome} \tag{16}$$

$$H_{cytosol} = \frac{(j_c + k_m^H)H_{out} + \frac{\alpha}{K_\alpha}C_{cytosol}(r = R_b) - k_m^H H_{carboxysome}}{k_m^H G + \frac{D}{R_b^2}}\left(\frac{D}{k_c R_c^2} + \frac{1}{R_C} - \frac{1}{r}\right) + H_{carboxysome} \tag{17}$$

Here the concentration of CO_2 at the cell membrane,

$$C_{cytosol}(r = R_b) = \frac{k_m^C C_{out} - (\frac{\alpha}{K_\alpha} + k_m^C)C_{carboxysome}}{(\frac{\alpha}{K_\alpha} + k_m^C)G + \frac{D}{R_b^2}}G + C_{carboxysome} \tag{18}$$

S2 Equations in carboxysome

In the carboxysome the equations are

$$\partial_t C = D\nabla^2 C + R_{CA} - R_{Rub} \tag{19}$$

$$\partial_t H = D\nabla^2 H - R_{CA}, \tag{20}$$

where the equation for the carbonic anhydrase reaction is

$$R_{CA}(H, C) = \frac{V_{ba}K_{ca}H - V_{ca}K_{ba}C}{K_{ba}K_{ca} + K_{ca}H + K_{ba}C} \tag{21}$$

Here V_{ba} and V_{ca} are the maximum rates of dehydration and hydration. K_{ba} and K_{ca} are the half maximum concentration rates for dehydration and hydration.

The equation for the RuBisCO reaction is

$$R_{Rub} = \frac{V_{max}C}{C + K_m} \tag{22}$$

$$K_m = K'_m(1 + \frac{O}{K_i}) \tag{23}$$

Here V_{max} is the maximum rate of carbon fixation by RuBisCO, and K_m is half maximum concentration rate, modified to include competitive binding with O_2, O.

We can use the solution in the cytosol to write a boundary condition at the carboxysome:

$$\begin{aligned}\frac{\partial C}{\partial r} &= -\frac{A_3}{r^2} \\ &= -\left(\frac{1}{R_c^2}\right)\frac{(\frac{\alpha}{K_\alpha} + k_m^C)C_{carboxysome} - k_m^C C_{out}}{(\frac{\alpha}{K_\alpha} + k_m^C)G + \frac{D}{R_b^2}}\end{aligned} \tag{24}$$

$$\begin{aligned}\frac{\partial H}{\partial r} &= -\frac{B_3}{r^2} \\ &= -\left(\frac{1}{R_c^2}\right)\frac{k_m^H H_{carboxysome} - (j_c + k_m^H)H_{out} - \frac{\alpha}{K_\alpha}C_{cytosol}(r = R_b)}{k_m^H G + \frac{D}{R_b^2}}\end{aligned} \tag{25}$$

S3 RuBisCO negligible in setting up CO_2 concentration

When RuBisCO negligible we can find the solution in the carboxysome as a balance between the carbonic anhydrase dehydration reaction and either the hydration reaction or diffusion.

S3.1 Carbonic anhydrase equilibrates carbon in carboxysome

If the carbonic anhydrase rate is faster than the diffusion rate then diffusion will be negligible and the solution in the carboxysome is set by $R_{CA} \approx 0$;

$$H_{carboxysome} \approx \frac{V_{ca}K_{ba}}{V_{ba}K_{ca}}C_{carboxysome}. \tag{26}$$

Another consequence of looking at equations 1.19 and 1.20 at steady state with $R_{Rub} \approx 0$ is that $\nabla^2(C + H) \approx 0$. Integrating once we get:

$$\begin{aligned}\frac{\partial(C + H)}{\partial r} &= \frac{a}{r^2} = 0 \\ \frac{\partial(C + H)}{\partial r}(r = R_c) &= 0\end{aligned} \tag{27}$$

the constant a must be zero, or else we would get a divergent solution at $r = 0$. This is the same as mass conservation. Since the RuBisCO reaction is negligible, the total flux of inorganic carbon in and out of the carboxysome must balance. Using boundary conditions 1.24 and 1.25 in equation 1.27, we find a second equation for H and C.

$$\begin{aligned}&\frac{(\frac{\alpha}{K_\alpha} + k_m^C)C_{carboxysome} - k_m^C C_{out}}{(\frac{\alpha}{K_\alpha} + k_m^C)G + \frac{D}{R_b^2}} + \frac{k_m^H H_{carboxysome} - (j_c + k_m^H)H_{out}}{k_m^H G + \frac{D}{R_b^2}} \\ &- \frac{\frac{\alpha}{K_\alpha}\frac{k_m^C C_{out} - (\frac{\alpha}{K_\alpha} + k_m^C)C_{carboxysome}}{(\frac{\alpha}{K_\alpha} + k_m^C)G + \frac{D}{R_b^2}}G + \frac{\alpha}{K_\alpha}C_{carboxysome}}{k_m^H G + \frac{D}{R_b^2}} = 0\end{aligned} \tag{28}$$

Using equation 1.55 we can find the CO_2 concentration in the carboxysome:

$$C_{carboxysome} = \frac{(j_c + k_m^H)H_{out}((k_m^C + \frac{\alpha}{K_\alpha})G + \frac{D}{R_b^2}) + k_m^C C_{out}((k_m^H + \frac{\alpha}{K_\alpha})G + \frac{D}{R_b^2})}{(k_m^C + \frac{\alpha}{K_\alpha})(1 + \frac{V_{ca}K_{ba}}{V_{ba}K_{ca}})k_m^H G + k_m^C(1 + \frac{k_m^H}{k_m^C}\frac{V_{ca}K_{ba}}{V_{ba}K_{ca}})\frac{D}{R_b^2}} \tag{29}$$

and the solution for H is set by 1.55.

S3.2 Low carbonic anhydrase concentration

It is possible for the hydration reaction to be negligible compared to diffusion when carbonic anhydrase becomes saturated. In this case $\nabla^2 H = \frac{Vba}{D}$ and $\nabla^2 C = -\frac{Vba}{D}$, implying

$$C = -\frac{V_{ba}}{6D_c}r^2 + A_1 \tag{30}$$

$$H = \frac{V_{ba}}{6D_c}r^2 + B_1. \tag{31}$$

applying boundary conditions 1.24 and 1.25 to these equations we find,

$$C_{carboxysome} = \frac{V_{ba}}{3D_c}R_c^3\left(G + \frac{D}{(\frac{\alpha}{K_\alpha} + k_m^C)R_b^2}\right) + \frac{V_{ba}}{6D_c}\left(R_c^2 - r^2\right) + \frac{k_m^C}{\frac{\alpha}{K_\alpha} + k_m^C}C_{out} \tag{32}$$

$$\begin{aligned} H_{carboxysome} = \frac{V_{ba}}{6D_c}(r^2 - R_c^2) - \frac{V_{ba}}{3D_c}R_c^3\left(G + \frac{D}{k_m^H R_b^2}\right) + \frac{(j_c + k_m^H)}{k_m^H}H_{out} \\ + \frac{\alpha}{k_m^H K_\alpha}\left(\frac{k_m^C C_{out}}{(\frac{\alpha}{K_\alpha} + k_m^C)G + \frac{D}{R_b^2}}\right) + \left(1 - \frac{(\frac{\alpha}{K_\alpha} + k_m^C)G}{(\frac{\alpha}{K_\alpha} + k_m^C)G + \frac{D}{R_b^2}}\right)\frac{\alpha}{k_m^H K_\alpha}C_{carboxysome} \end{aligned} \tag{33}$$

We have differentiated the diffusion constant in the carboxysome, D_c from the one in the cytosol, D. At the carboxysome shell we use the same diffusion constant as in the cytosol, since this is how we calculated the carboxysome permeability in the text.

S4 RuBisCO significant

If RuBisCO is significant then the approximation 1.27 doesn't hold. Instead we need to loosen the condition. Instead of the flux of $H + C$ out of the carboxysome being zero, it must be equal to the amount of CO_2 consumed in the carboxysome.

$$\int D\frac{\partial(C+H)}{\partial r}(r = R_c)dS_{carboxysome} = \int \frac{V_{max}C_{carboxysome}}{C_{carboxysome} + K_m}dV_{carboxysome} \tag{34}$$

$$4\pi R_c^2 D(-\frac{A_3}{R_c^2} - \frac{B_3}{R_c^2}) = \frac{4}{3}\pi R_c^3\frac{V_{max}C_{carboxysome}}{C_{carboxysome} + K_m} \tag{35}$$

$$A_3 + B_3 = -\frac{R_c^3}{3D}\frac{V_{max}C_{carboxysome}}{C_{carboxysome} + K_m} \tag{36}$$

with A_3 and B_3 defined as before:

$$A_3 = \frac{(\frac{\alpha}{K_\alpha} + k_m^C)C_{carboxysome} - k_m^C C_{out}}{(\frac{\alpha}{K_\alpha} + k_m^C)G + \frac{D}{R_b^2}} \tag{37}$$

$$B_3 = \frac{(k_m^H H_{carboxysome} - (j_c + k_m^H)H_{out} - \frac{\alpha}{K_\alpha}C_{cytosol}(r = R_b))}{k_m^H G + \frac{D}{R_b^2}} \tag{38}$$

We still use the fact that the carboxysome is small, and so diffusion equilibrates the concentration across the carboxysome very quickly. The equation for HCO_3^- therefore, still enforces the ratio $H_{carboxysome} \approx \frac{V_{ca}K_{ba}}{V_{ba}K_{ca}}C_{carboxysome}$.

If we define the following:

$$N = (j_c + k_m^H)H_{out}((k_m^C + \frac{\alpha}{K_\alpha})G + \frac{D}{R_b^2}) + k_m^C C_{out}((k_m^H + \frac{\alpha}{K_\alpha})G + \frac{D}{R_b^2}) \tag{39}$$

$$M = (k_m^C + \frac{\alpha}{K_\alpha})(1 + \frac{V_{ca}K_{ba}}{V_{ba}K_{ca}})k_m^H G + k_m^C(1 + \frac{k_m^H}{k_m^C}\frac{V_{ca}K_{ba}}{V_{ba}K_{ca}})\frac{D}{R_b^2} \tag{40}$$

$$P = ((\frac{\alpha}{K_\alpha} + k_m^C)G + \frac{D}{R_b^2})(k_m^H G + \frac{D}{R_b^2}) \tag{41}$$

then we can rewrite equation 1.36

$$N - MC_{carboxysome} - \frac{R_c^3 V_{max} C_{carboxysome}}{3D(C_{carboxysome} + K_m)}P = 0 \tag{42}$$

$$C_{carboxysome}^2 + (K_m - \frac{N}{M} + \frac{R_c^3 V_{max} P}{3DM})C_{carboxysome} - K_m\frac{N}{M} = 0 \tag{43}$$

$$C_{carboxysome} = \frac{1}{2}(\frac{N}{M} - \frac{R_c^3 V_{max} P}{3DM} - K_m) \pm \frac{1}{2}\sqrt{(\frac{N}{M} - \frac{R_c^3 V_{max} P}{3DM} - K_m)^2 + 4\frac{N}{M}K_m} \tag{44}$$

For comparison, when RuBisCO is not significant the solution, equation 1.29, can be written as

$$C_{carboxysome} = \frac{N}{M}. \tag{45}$$

When RuBisCO is significant but saturated, and the reaction is constant, we can make the approximation:

$$C_{carboxysome} = \frac{N}{M} - \frac{R_c^3 V_{max} P}{3MD}. \tag{46}$$

When RuBisCO is significant but unsaturated, and the reaction is linear in CO_2 concentration, we can make the approximation:

$$C_{carboxysome} = \frac{N}{M + \frac{R_c^3 V_{max} P}{3K_m D}}. \tag{47}$$

S5 Reactions everywhere in cell

When RuBisCO and carbonic anhydrase are everywhere in the cell the equations previously used in the carboxysome apply everywhere:

$$\partial_t C = D\nabla^2 C + R_{CA} - R_{Rub} \tag{48}$$

$$\partial_t H = D\nabla^2 H - R_{CA}, \tag{49}$$

$$R_{CA}(H, C) = \frac{V_{ba}K_{ca}H - V_{ca}K_{ba}C}{K_{ba}K_{ca} + K_{ca}H + K_{ba}C} \tag{50}$$

$$R_{Rub} = \frac{V_{max} C}{C + K_m} \tag{51}$$

but now the only boundary condition is at the cell membrane;

$$D\frac{\partial C}{\partial r} = -\frac{\alpha C_{cytosol}}{K_\alpha + C_{cytosol}} + k_m^C \left(C_{out} - C_{cytosol}\right) \tag{52}$$

$$\begin{aligned} D\frac{\partial H}{\partial r} = j_c H_{out} + &\frac{\alpha C_{cytosol}}{K_\alpha + C_{cytosol}} \\ &+ k_m^H \left(H_{out} - H_{cytosol}\right) \end{aligned} \tag{53}$$

Equations 1.48 and 1.49 can now be solved making the same approximations as before.

S5.1 Carbonic anhydrase equilibrates carbon in cell

Using the analogous approximation to equation 1.27, set by boundary conditions 1.53 and 1.53,

$$\frac{\partial (C + H)}{\partial r}(r = R_b) = 0 \tag{54}$$

and

$$H_{cytosol} \approx \frac{V_{ca} K_{ba}}{V_{ba} K_{ca}} C_{cytosol}. \tag{55}$$

we find

$$\begin{aligned} &-\frac{\alpha C_{cytosol}}{K_\alpha + C_{cytosol}} + k_m^C \left(C_{out} - C_{cytosol}\right) \\ &+ j_c H_{out} + \frac{\alpha C_{cytosol}}{K_\alpha + C_{cytosol}} + k_m^H \left(H_{out} - \frac{V_{ca} K_{ba}}{V_{ba} K_{ca}} C_{cytosol}\right) = 0 \end{aligned} \tag{56}$$

$$C_{cytosol} = \frac{k_m^C C_{out} + (j_c + k_m^H) H_{out}}{k_m^C (1 + \frac{k_m^H}{k_m^C} \frac{V_{ca} K_{ba}}{V_{ba} K_{ca}})} \tag{57}$$

S5.2 Low carbonic anhydrase concentration

When carbonic anhydrase concentration is low and saturated we have the same equations as before,

$$C = -\frac{V_{ba}}{6D} r^2 + A_1 \tag{58}$$

$$H = \frac{V_{ba}}{6D} r^2 + B_1. \tag{59}$$

and apply boundary conditions 1.52 and 1.53. We get:

$$C_{cytosol} = \frac{k_m^C C_{out}}{\frac{\alpha}{K_\alpha} + k_m^C} + V_{ba}\left(\frac{R_b}{3(\frac{\alpha}{K_\alpha} + k_m^C)} + \frac{R_b^2}{6D} - \frac{r^2}{6D}\right) \tag{60}$$

$$H_{cytosol} = V_{ba}\left(\frac{r^2}{6D} - \frac{R_b^2}{6D} - \frac{R_b}{3k_m^H}\right) + \frac{j_c + k_m^H}{k_m^H} H_{out} + \frac{\alpha}{K_\alpha k_m^H} C_{cytosol}(r = R_b) \tag{61}$$

S5.3 RuBisCO significant

Following the same methodology as in section S3, we write

$$\int D\frac{\partial(C+H)}{\partial r}|_{r=R_B}dS_{cell} = \int \frac{V_{max}C}{C+K_m}dV_{cell} \tag{62}$$

$$4\pi R_b^2 D\left(\frac{\partial C}{\partial r}|_{r=R_b} + \frac{\partial H}{\partial r}|_{r=R_b}\right) = \frac{4}{3}\pi R_b^3 \frac{V_{max}C}{C+K_m} \tag{63}$$

$$D\frac{\partial C}{\partial r}|_{r=R_b} + D\frac{\partial H}{\partial r}|_{r=R_b} = \frac{R_b}{3}\frac{V_{max}C}{C+K_m} \tag{64}$$

Where the two partial derivatives at $r = R_b$ are defined by boundary conditions 1.52 and 1.53. We can define

$$N_2 = k_m^C C_{out} + (j_c + k_m^H)H_{out} \tag{65}$$

$$M_2 = k_m^C\left(1 + \frac{k_m^H}{k_m^C}\frac{V_{ca}K_{ba}}{V_{ba}K_{ca}}\right) \tag{66}$$

Note that N_2 and M_2 do not have the same dimensions as N and M defined previously. Using these definitions we can solve for the CO_2 concentration in the cytosol.

$$N_2 - M_2 C - \frac{R_b}{3}\frac{V_{max}C}{C+K_m} = 0 \tag{67}$$

$$C_{cytosol}^2 + (K_m - \frac{N_2}{M_2} + \frac{R_b V_{max}}{3M_2})C_{cytosol} - K_m\frac{N_2}{M_2} = 0 \tag{68}$$

$$\begin{aligned} C_{cytosol} &= \frac{1}{2}\left(\frac{N_2}{M_2} - \frac{R_b V_{max}}{3M_2} - K_m\right) \\ &+\frac{1}{2}\sqrt{(K_m - \frac{N_2}{M_2} + \frac{R_b V_{max}}{3M_2})^2 + \frac{4K_m N_2}{M_2}} \end{aligned} \tag{69}$$

The equation for CO_2 concentration assuming negligible RuBisCO activity, equation 1.57, can be rewritten:

$$C_{cytosol} = \frac{N_2}{M_2} \tag{70}$$

If RuBisCO is saturated then the concentration is described by:

$$C = \frac{N_2}{M_2} - \frac{R_b}{3}V_{max} \tag{71}$$

If RuBisCo is unsaturated:

$$C = \frac{N_2}{M_2 + \frac{R_b V_{max}}{3K_m}} \tag{72}$$

S6 Reactions localized without carboxysome

We assume the region where the enzymes are located remains the same size. This could be accomplished by attaching the enzymes to a scaffold instead of incapsulating them.

When there is no carboxysome shell, the boundary condition at R_c changes to:

$$C_{cytosol} = C_{scaffold} \tag{73}$$

$$H_{cytosol} = H_{scaffold} \tag{74}$$

where the concentration in the cytosol is the same as before.

$$C_{cyto} = \frac{A_3}{r} + A_4 = A_3\left(\frac{1}{r} - \frac{1}{R_c}\right) + C_{scaffold} \tag{75}$$

$$H_{cyto} = \frac{B_3}{r} + B_4 = B_3\left(\frac{1}{r} - \frac{1}{R_c}\right) + H_{scaffold} \tag{76}$$

Plugging equations 1.75 and 1.76 into the following boundary conditions at the cell membrane.

$$D\frac{\partial C}{\partial r} = -\frac{\alpha C_{cytosol}}{K_\alpha + C_{cytosol}} + k_m^C\left(C_{out} - C_{cytosol}\right) \tag{77}$$

$$\begin{aligned} D\frac{\partial H}{\partial r} = j_c H_{out} + &\frac{\alpha C_{cytosol}}{K_\alpha + C_{cytosol}} \\ &+ k_m^H\left(H_{out} - H_{cytosol}\right) \end{aligned} \tag{78}$$

we find the values of A_3 and B_3 are the same as before, but with a different definition of G.

$$A_3 = \frac{(\frac{\alpha}{K_\alpha} + k_m^C)C_{scaffold} - k_m^C C_{out}}{(\frac{\alpha}{K_\alpha} + k_m^C)G + \frac{D}{R_b^2}} \tag{79}$$

$$B_3 = \frac{(k_m^H H_{scaffold} - (j_c + k_m^H)H_{out} - \frac{\alpha}{K_\alpha}C_{cytosol}(r = R_b))}{k_m^H G + \frac{D}{R_b^2}} \tag{80}$$

$$G = \left(\frac{1}{R_c} - \frac{1}{R_b}\right) \tag{81}$$

$$C_{cytosol} = \frac{k_m^C C_{out} - (\frac{\alpha}{K_\alpha} + k_m^C)C_{scaffold}}{(\frac{\alpha}{K_\alpha} + k_m^C)G + \frac{D}{R_b^2}}\left(\frac{1}{R_C} - \frac{1}{r}\right) + C_{scaffold} \tag{82}$$

$$\begin{aligned} H_{cytosol} =& \frac{(j_c + k_m^H)H_{out} + \frac{\alpha}{K_\alpha}C_{cytosol}(r = R_b) - k_m^H H_{scaffold}}{k_m^H G + \frac{D}{R_b^2}}\left(\frac{1}{R_C} - \frac{1}{r}\right) \\ &+ H_{scaffold} \end{aligned} \tag{83}$$

Here the concentration of CO_2 at the cell membrane,

$$C_{cytosol}(r = R_b) = \frac{k_m^C C_{out} - (\frac{\alpha}{K_\alpha} + k_m^C)C_{scaffold}}{(\frac{\alpha}{K_\alpha} + k_m^C)G + \frac{D}{R_b^2}}G + C_{scaffold} \tag{84}$$

The solution in the carboxysome is exactly what we have calculated before, in either the carbonic anhydrase equilibrating or saturated case, except with our new definition of G.
